# Mixed‐Potential‐Driven Catalysis: An Electrochemical Mechanism for Room‐Temperature CO Oxidation on Gold Catalysts

**DOI:** 10.1002/advs.202505994

**Published:** 2025-06-25

**Authors:** Mo Yan, Muhammad Asif, Ravi Singh, Kotaro Takeyasu, Junji Nakamura

**Affiliations:** ^1^ International Institute for Carbon‐Neutral Energy Research (I2CNER) Kyushu University 744 Motooka, Nishi‐ku, Fukuoka‐shi Fukuoka 819‐0395 Japan; ^2^ Graduate School of Science and Technology University of Tsukuba 1‐1‐1 Tennodai Tsukuba Ibaraki 305–8573 Japan; ^3^ Institute of Pure and Applied Sciences University of Tsukuba 1‐1‐1 Tennodai Tsukuba Ibaraki 305–8573 Japan; ^4^ Tsukuba Research Centre for Energy and Materials Science University of Tsukuba 1‐1‐1 Tennodai Tsukuba Ibaraki 305–8573 Japan; ^5^ R&D Center for Zero CO2 Emission with Functional Materials University of Tsukuba 1‐1‐1 Tennodai Tsukuba Ibaraki 305–8573 Japan; ^6^ Institute for Catalysis Hokkaido University Kita21, Nishi10, Kita‐ku Sapporo Hokkaido 001–0021 Japan

**Keywords:** CO oxidation, electrochemical half‐reaction, gold catalyst, heterogeneous catalysis, mixed potential

## Abstract

Gold is an effective catalyst for low‐temperature CO oxidation, yet its mechanism remains debated. Among the various proposed mechanisms, increasing attention has been given to a possible electrochemical pathway in recent studies. Here, experimental evidence is reported that CO oxidation at room temperature in electrolytes proceeds via coupled electrochemical CO oxidation and oxygen reduction half‐reactions, i.e., the mixed‐potential‐driven mechanism. A model system is designed with deposited Au nanoparticles (NPs) and nitrogen‐doped reduced graphene oxide (NrGO) on carbon paper acting as spatially separated electrodes in a single electrolysis cell. After shorting the two electrodes, the reaction current and mixed potential of the overall CO oxidation are measured without any externally applied bias. Independent current–potential curves for each half‐reaction are also obtained under identical experimental conditions. The intersection points of these curves, representing the predicted mixed potential and net current, showed good agreement with the values measured in the short‐circuited system. These results provide clear evidence that CO oxidation in aqueous media proceeds via a mixed‐potential‐driven mechanism, analogous to corrosion. This finding provides strong support for the hypothesis that low‐temperature CO oxidation over‐supported Au catalysts in the presence of water vapor proceeds via a similar electrochemical pathway in gas‐phase heterogeneous catalysis.

## Graphical abstract

Gold catalyzes low‐temperature CO oxidation, yet its mechanism is debated. A model system is designed to verify that it proceeds via a mixed‐potential‐driven reaction mechanism involving coupled electrochemical CO oxidation half‐reaction and O_2_ reduction half‐reaction. After shorting the two electrodes, the reaction current and mixed potential of the overall CO oxidation are measured without any bias potential.

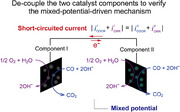

## 1 Introduction

The catalytic oxidation of carbon monoxide (CO) is of both practical and fundamental importance, with broad applications in pollution control, gas sensing, and energy conversion technologies.^[^ ^1^ ^]^ Moreover, it has long served as a prototypical reaction for exploring fundamental concepts in heterogeneous catalysis.^[^ ^2^, ^3^, ^4^ ^]^ Gold (Au), regarded as chemically inert,^[^ ^5^ ^]^ has been shown to exhibit remarkable catalytic activity when dispersed as nanoparticles (NPs) on suitable supports, particularly for low‐temperature CO oxidation (CO + 1/2 O_2_ → CO_2_).^[^ ^6^ ^]^ Despite extensive mechanistic investigations, a unified framework that accounts for the intricate interplay among catalyst support,^[^ ^7^ ^]^ Au particle size,^[^ ^8^ ^]^ and the metal–support interfacial perimeter^[^ ^9^ ^]^ remains elusive. These factors are all known to critically influence catalytic performance. One particularly intriguing and yet unresolved phenomenon is the pronounced promotional effect of water on CO oxidation activity, both in gas phase and liquid phase systems.^[^ ^10^, ^11^, ^12^, ^13^, ^14^, ^15^ ^]^ Numerous studies have attempted to elucidate this effect mechanistically.^[^ ^11^, ^12^, ^13^, ^14^, ^15^, ^16^, ^17^, ^18^, ^19^, ^20^, ^21^, ^22^, ^23^, ^24^ ^]^ The high activity in humid environments and liquid conditions suggests the involvement of electrochemical pathways.^[^ ^25^ ^]^ Indeed, it is well known that CO can be electrochemically oxidized on Au surfaces, and the size‐dependent activity of Au on TiO_2_ observed in heterogeneous catalysis has also been reported in electrochemical CO oxidation systems.^[^ ^26^, ^27^, ^28^, ^29^ ^]^

**Scheme 1:**
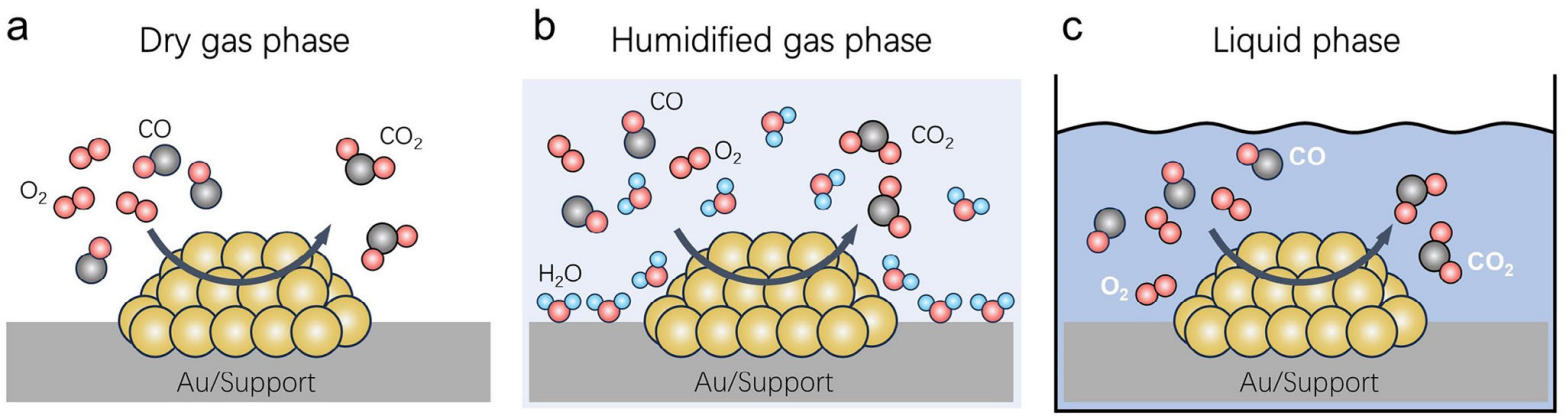
Illustration of three reaction conditions for CO oxidation over supported Au catalysts. a) In the dry gas phase, CO and O_2_ interact with the Au/support catalyst in the absence of water. b) In the humidified gas phase, water vapor may adsorb onto the catalyst surface or condense locally, forming nanoscale aqueous films that enable limited ionic conductivity, depending on the partial pressure of H_2_O and surface structure. c) In the liquid phase, a continuous aqueous medium facilitates effective ionic conduction throughout the system.

To explore this hypothesis, we analyzed previous reports on CO oxidation over Au catalysts by classifying them into three distinct regimes: i) dry gas phase in the absence of water vapor, ii) gas phase under humidified conditions, and iii) liquid phase, as illustrated in **Scheme** **1**. The catalytic activities were quantitatively compared using turnover frequency (TOF) as a normalized metric, with detailed data provided in (Figure S5 and Tables S3–S5, Supporting Information). While the reaction temperatures across these studies were generally near room temperature, slight differences in the partial pressures of CO and O_2_ were noted. Notably, for carbon‐supported Au catalysts in the liquid phase, the reported TOF values are significantly higher than those under dry gas phase conditions,^[^ ^13^, ^15^ ^]^ despite the inherently low solubility of CO in water. This striking enhancement suggests the operation of a distinct reaction pathway involving electrochemical mechanisms. For Au/TiO_2_ catalysts, which have been extensively studied under humidified conditions, the desorption temperature of adsorbed water lies between 250 and 400 K, depending on the surface structure.^[^ ^30^ ^]^ Given that CO oxidation was carried out near room temperature in these systems, a substantial amount of water is expected to remain adsorbed on the TiO_2_ surface. This implies that water may act as a proton‐conducting medium, facilitating electrochemical interactions between the Au particles and the TiO_2_ support. In addition, promotional effects of alkali species have been reported in both gas‐phase and liquid‐phase CO oxidation systems,^[^ ^13^, ^14^, ^15^, ^31^, ^32^, ^33^ ^]^ further supporting the involvement of ionic or electrochemical contributions.

In light of these findings, we propose that CO oxidation over supported Au catalysts in aqueous electrolytes or humidified environments proceeds via a mixed‐potential‐driven mechanism. In this mechanism, oxidation and reduction half‐reactions occur at spatially distinct sites on the catalyst, similar to corrosion phenomena, and are electrically coupled through short‐circuiting. There is a growing recognition that many thermochemical catalytic processes involving water may proceed via two electrochemically coupled half‐reactions. Such corrosion‐like mechanisms have now been identified in a wide variety of heterogeneous catalytic reactions,^[^ ^34^, ^35^, ^36^, ^37^, ^38^, ^39^, ^40^, ^41^, ^42^, ^43^ ^]^ and direct electron transfer evidence between distinct catalyst components has been reported.^[^ ^44^, ^45^, ^46^, ^47^, ^48^ ^]^ We refer to this class of reactions as mixed‐potential‐driven catalysis, for which we have also recently developed a theoretical framework.^[^ ^49^ ^]^ In the absence of any externally applied potential, the catalyst system in the aqueous phase experiences an intrinsic overpotential derived from the Gibbs free energy difference between the anodic and cathodic half‐reactions.^[^ ^49^ ^]^ In essence, the catalyst operates like a nanoscale galvanic cell under short‐circuit conditions.^[^ ^44^, ^49^ ^]^ The key distinction from a conventional galvanic cell is that, in mixed‐potential‐driven catalysis, all catalyst components are exposed to the same reactants. Here, the relative catalytic activities for the oxidation and reduction half‐reactions determine the direction of electron flow, thereby defining which component acts as the anode or cathode.

Specifically, CO oxidation may proceed via a mixed‐potential‐driven mechanism^[^ ^50^ ^]^ involving the coupling of the electrochemical CO oxidation reaction (COOR) and O_2_ reduction reaction (ORR), mediated by either OH^−^ or H^+^.

(1) $$\mathrm{CO}+2OH^{-}\to CO_{2}+H_{2}O+2e^{-}$$

(2) $$\frac{1}{2}O_{2}+H_{2}O+2e^{-}\to 2OH^{-}$$

(3) $$\mathrm{CO}+H_{2}O\to CO_{2}+2H^{+}+2e^{-}$$

(4) $$\frac{1}{2}O_{2}+2H^{+}+2e^{-}\to H_{2}O$$

**Figure 1:**
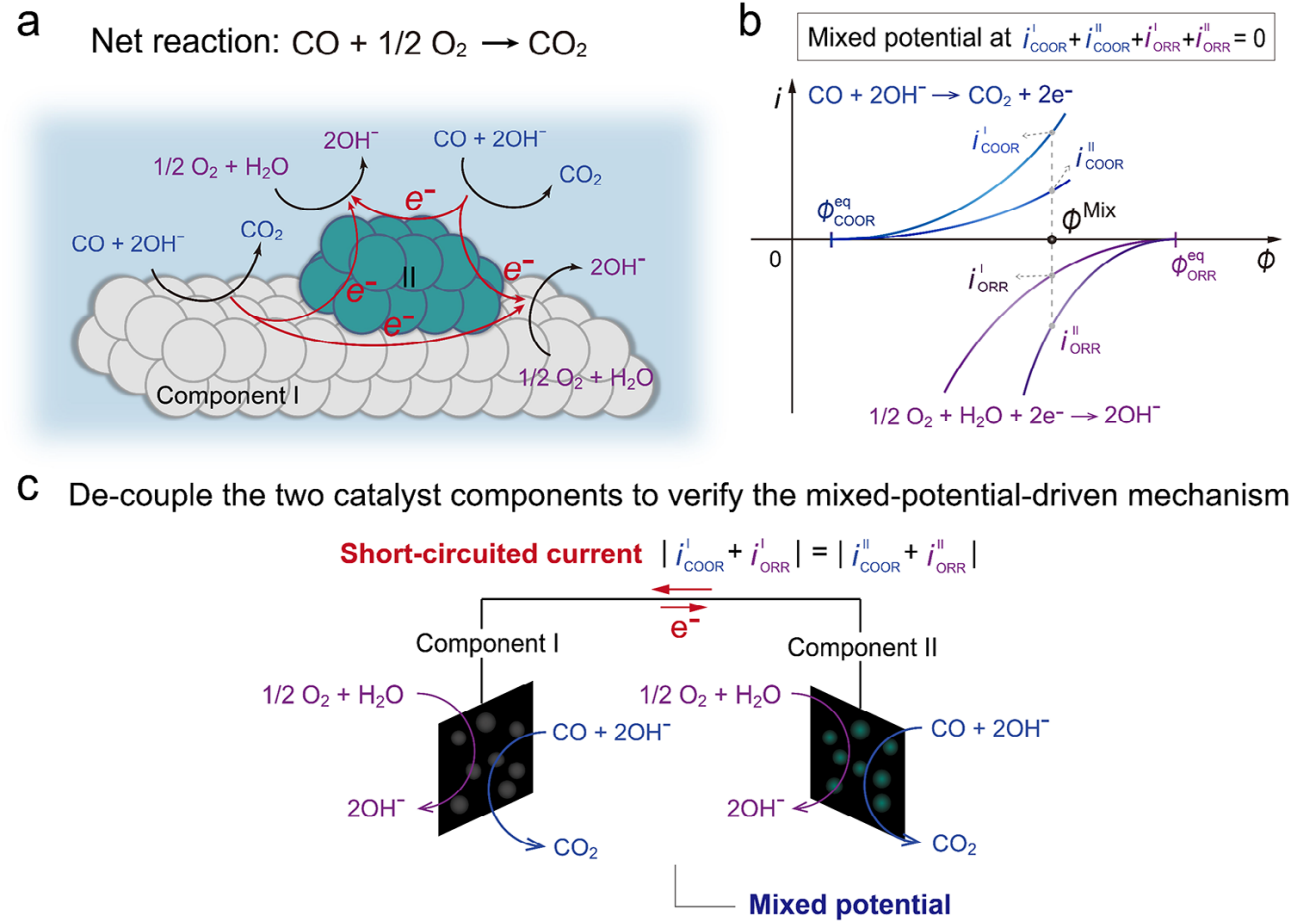
A proposed mixed‐potential‐driven mechanism for the room‐temperature CO oxidation in the aqueous phase. a) A schematic of the mixed‐potential‐driven CO catalytic reaction occurring on the bicomponent catalyst. CO oxidation reaction (COOR) and oxygen reduction reaction (ORR) can simultaneously occur in both catalyst components I and II. Electrons are transferred within the catalysts without any external applied potential. We exemplify the hydroxyl group as the reactant, but H_2_O is also possible, as described by Equations (3) and (4). b) Schematic illustration of the four independent current–potential curves for the COOR and ORR on components I and II. The mixed potential is located at which the sum of the four reaction currents is zero. $\phi_{\mathrm{COOR}}^{\mathrm{eq}}$ and $\phi_{\mathrm{ORR}}^{\mathrm{eq}}$ are the equilibrium potentials of COOR and ORR, respectively. c) Schematic representation of the electrolysis cell for short‐circuited experiments. By de‐coupling the two components, the apparent reaction current flows between two electrodes via an external circuit and the mixed potential can be measured simultaneously. Both electrodes are exposed to identical gas/liquid environments. No external potential is applied during operation.

**Figure** **1**a presents a conceptual model of mixed‐potential‐driven CO oxidation taking place on a bicomponent catalyst. In this model, both catalyst components I and II are exposed to identical reactants, CO and O_2_, and can carry out both COOR and ORR half‐reactions. The direction of electron or current flow is governed by the relative catalytic activities of components I and II toward COOR and ORR. Figure 1b presents a schematic illustration of the independent current–potential curves for each half‐reaction in Figure 1a. The mixed potential ($\phi^{\mathrm{Mix}}$) is the point where the currents of the COOR are equal in magnitude to the currents of the ORR.^[^ ^50^ ^]^ $\phi_{\mathrm{COOR}}^{\mathrm{eq}}$ and $\phi_{\mathrm{ORR}}^{\mathrm{eq}}$ are the equilibrium potentials of COOR and ORR, respectively. The overpotentials, $\phi^{\mathrm{Mix}}-\phi_{\mathrm{COOR}}^{\mathrm{eq}}$ and $\phi^{\mathrm{Mix}}-\phi_{\mathrm{ORR}}^{\mathrm{eq}}$, are thus applied to accelerate COOR and ORR, respectively.

The aim of this study is to verify the mixed‐potential‐driven mechanism for Au‐catalyzed CO oxidation in liquid electrolytes by using a model catalytic system. As illustrated in Figure 1c, the model consists of two distinct catalytic components (I and II) electrically connected via an external circuit. This configuration allows for the direct detection of electron transfer between the catalyst components and simultaneous measurement of the mixed potential. A similar method has been successfully applied in our previous study to demonstrate the mixed potential mechanism in the oxidation of glucose.^[^ ^45^ ^]^ The current–potential characteristics of each half‐reaction, COOR, and ORR, on components I and II are then independently evaluated in order to confirm the consistency with the measured mixed potential and short‐circuited current in Figure 1c.^[^ ^34^, ^35^, ^36^, ^38^, ^40^, ^43^ ^]^

## 2 Results and Discussion

### 2.1 Verifying the Mixed‐Potential‐Driven CO Oxidation by Short‐Circuited Experiments

**Figure 2:**
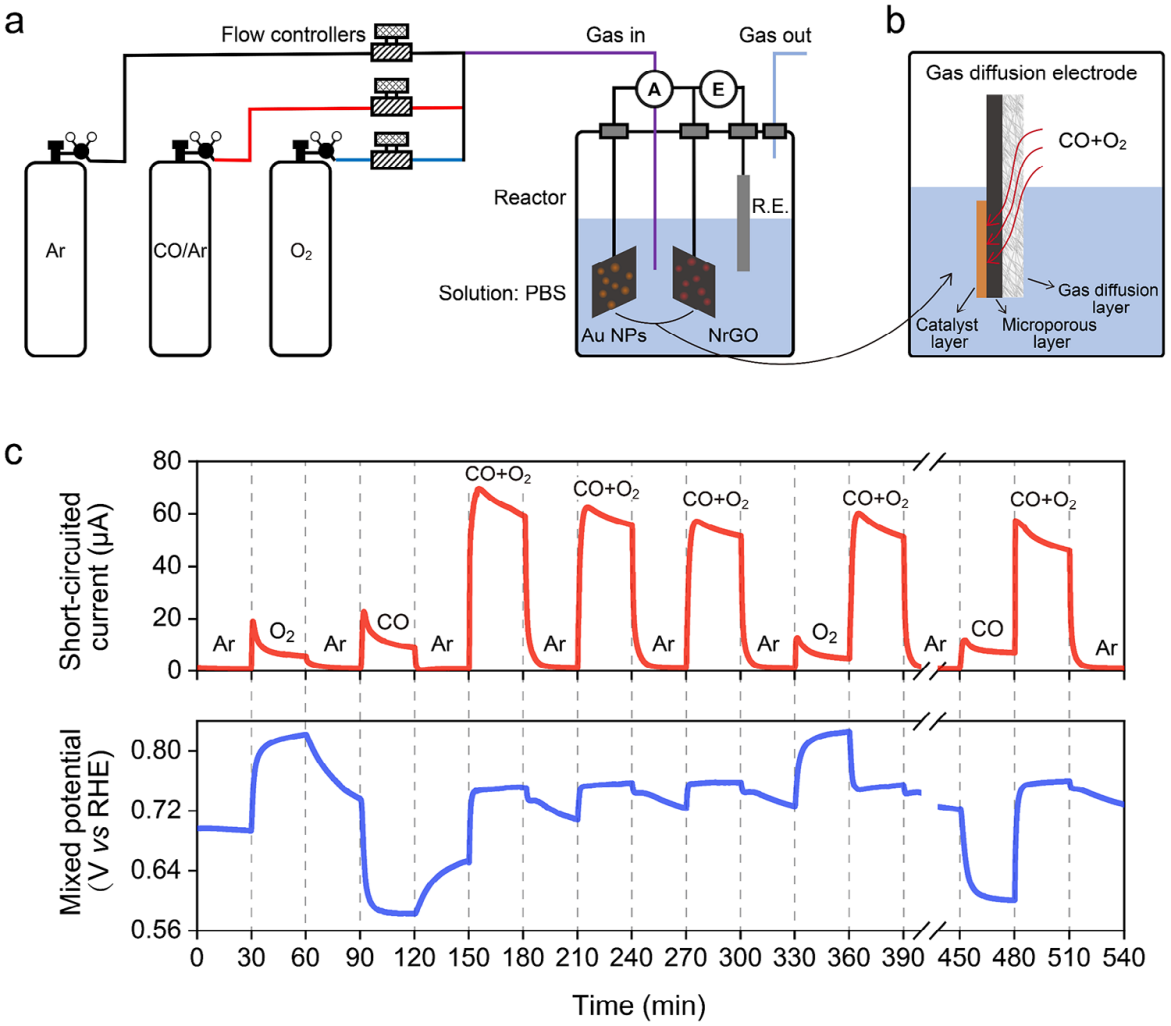
Short‐circuited current and mixed potential measurements. a) A schematic of the gas flow and the reactor used for short‐circuited experiments. The desired pressures of CO and O_2_ (adjusted by the flow rate of CO (5%)/Ar and ultrahigh‐purity O_2_) were continuously introduced into the single cell. Gas flow rates were controlled by mass flow controllers. Au NPs on carbon paper and NrGO on carbon paper were employed as the macroscopic gas diffusion electrodes and externally short‐circuited. Two potentiostats were employed to simultaneously monitor the short‐circuited current and mixed potential. There was no external applied potential, and both the short‐circuited Au NPs and NrGO electrodes were under identical gas/liquid environments during operation. b) Schematic of the gas diffusion electrode (GDE) architecture used in this study. The GDE consists of a catalyst layer, a microporous carbon layer, and a gas diffusion layer (carbon cloth). This structure allows CO and O_2_ to diffuse directly through the gas channels to the catalyst/electrolyte interface, enhancing mass transport and minimizing limitations from gas solubility in the liquid phase. c) The short‐circuited current and mixed potential are monitored simultaneously at a periodic switch between Ar, CO (0.04 atm), O_2_ (0.2 atm), and CO (0.04 atm) + O_2_ (0.2 atm) feeds in the single cell. Reaction conditions: 0.25 M PBS (15 mL, pH 7.2); 25 ± 1 °C; total flow rate: 50 cm^−3^ min^−1^.

To directly prove that CO oxidation can proceed via the mixed‐potential‐driven mechanism, it is necessary to measure the short‐circuited current and CO_2_ products and show that their stoichiometric relationship is established. We thus constructed a setup to monitor the short‐circuited currents and mixed potentials for room‐temperature CO oxidation in the aqueous phase (**Figure** **2**a,b). For the two catalyst components of Figure 1, the Au NPs supported on carbon catalyst is not active for gas‐phase CO oxidation at a low temperature of 300 K, but show good activity on immersion in aqueous solutions.^[^ ^15^, ^25^ ^]^ We thus selected Au NPs as one component for the COOR (Equation 1 or 3) and nitrogen‐doped reduced graphene oxide (NrGO) as the other component for the ORR (Equation 2 or 4). NrGO exhibits high activity for ORR even in the presence of CO.^[^ ^51^, ^52^ ^]^ The Au NPs and NrGO were fixed on a hydrophobic carbon‐based gas diffusion layer (GDL) as electrodes (catalyst synthesis, characterization, and electrode fabrication details in Figures S1–S3, as well as Tables S1 and S2, Supporting Information). This architecture enabled efficient gas transport to the catalyst layer, avoiding the low solubility of CO and O_2_ in water. In addition, a phosphate buffer solution (PBS) was added to the reactor to keep the pH constant and to act as an electrolyte.

Throughout the reaction, the short‐circuited currents between the Au NPs and the NrGO electrodes were monitored by a potentiostat. Simultaneously, the mixed potentials were measured by the other potentiostat at an open circuit potential relative to a reference electrode. X‐ray photoelectron spectroscopy (XPS) indicated that almost no Au oxidation occurred after the reaction (Figure S2, Supporting Information). In separate experiments, we quantitatively analyzed CO_2_‐derived products in short‐circuited and non‐short‐circuited conditions, such as bicarbonate, dissolved in aqueous solutions by UV–vis spectroscopy.

Figure 2c shows that short‐circuited currents flowed and the mixed potential changed simultaneously when CO + O_2_ gas was introduced to the reactor. The current flows from the NrGO electrode to the Au NPs electrode, which means that mainly the reduction half‐reactions at the NrGO electrode and the oxidation half‐reactions at the Au NPs electrode are dominant. Small currents were also observed with CO only and O_2_ only, but these were found to be due to the reduction and oxidation of the electrodes themselves by control experiments (Figures S7–S10, see Sections S2–S5, Supporting Information for detailed discussion). Importantly, a much higher short‐circuited current was generated when the feed was switched to the mixture gas of CO + O_2_, while the measured mixed potential ($\phi_{\mathrm{Mea}}^{\mathrm{Mix}}$) of a mixture CO + O_2_ atmosphere located at ≈0.75 V (all potentials were referenced to the RHE). The gradual decline in short‐circuited current upon CO + O_2_ introduction in Figure 2c and Figure S6 (Supporting Information) could be attributed to the transport limitation of CO, as discussed later. Electrolyte flooding and salt precipitation in the carbon‐based gas diffusion layer may degrade its hydrophobicity and then obstruct gas access to the catalyst, which is consistent with similar observations in gas‐fed electrochemical systems.^[^ ^48^, ^53^, ^54^, ^55^, ^56^ ^]^ Additionally, carbonate species formed during CO oxidation may accumulate on the surface and block active sites.^[^ ^57^, ^58^, ^59^ ^]^ We thus consider that the decrease of current does not necessarily indicate a loss of catalytic activity. Repeated switching between an Ar atmosphere and a mixture of CO + O_2_ atmosphere in the single cell led to concomitant switching of the short‐circuited current between approximately zero and 70 µA. The short‐circuited current for the mixture gas of CO + O_2_ provided direct evidence of the electron transfer in the room‐temperature CO oxidation in the aqueous phase.

### 2.2 Independent Current–Potential Behaviors Predict the Mixed Potential and Short‐Circuited Current

**Figure 3:**
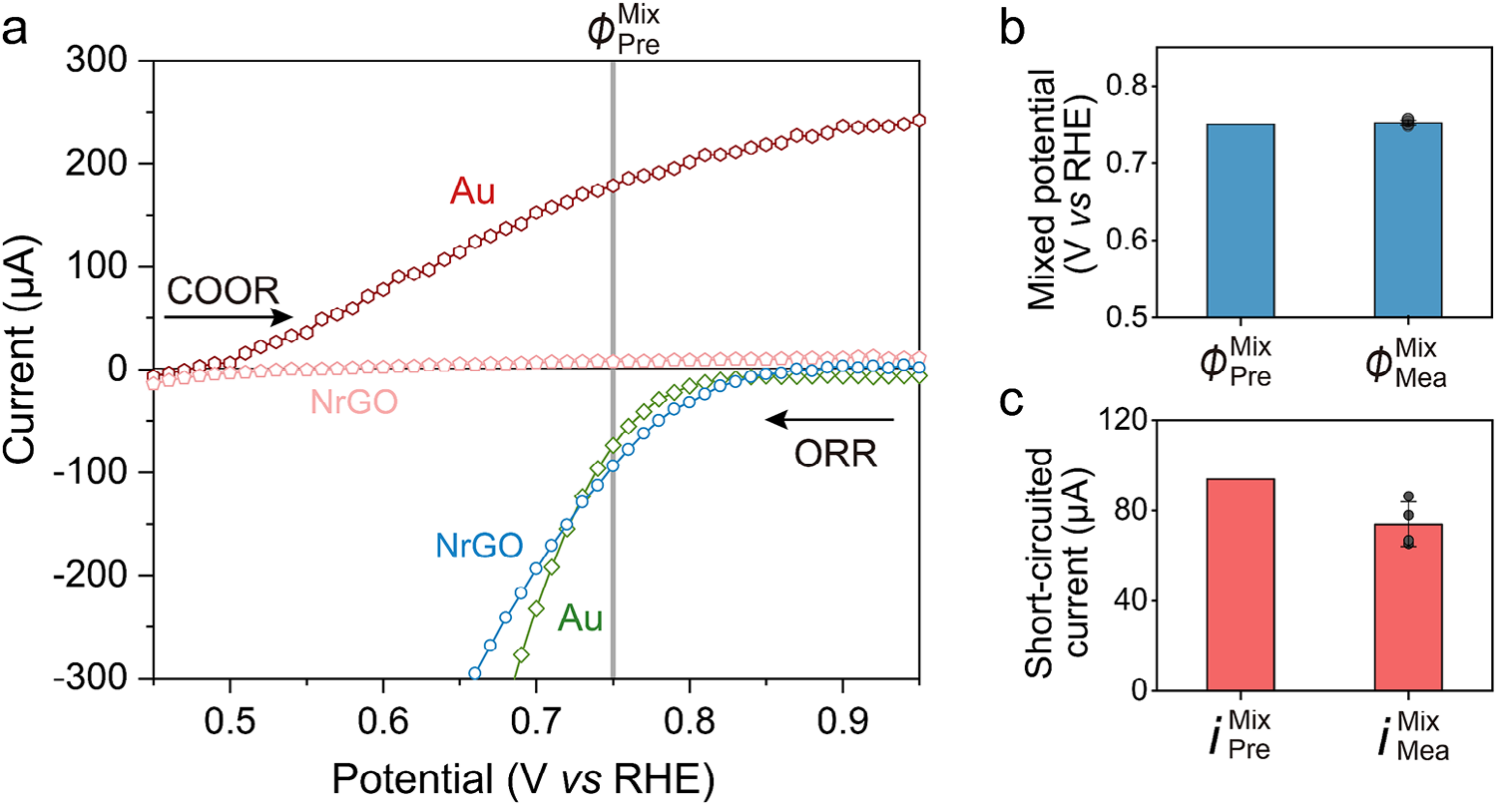
The mixed potential and short‐circuited current are predicted from the current–potential behaviors for COOR and ORR. a) Current–potential curves of COOR and ORR for deposited Au NPs and NrGO electrodes. The COOR is conducted in the absence of O_2_, while the ORR is collected without CO. The arrows indicate the direction of the scan. Both the COOR and ORR curves were corrected by the background currents which were obtained under 1 atm Ar. The mixed potential at which the sum of the currents of COOR and ORR is zero is marked by the gray line. Reaction conditions: 0.25 m pbs (pH 7.2); 25 ± 1 °C; 0.04 atm CO for COOR; 0.2 atm O_2_ for ORR. b,c) Comparison between predicted (from current–potential curves) and measured (from short‐circuited experiments) mixed potential (b) and reaction current (c). The error bar represents the standard deviation of four replicate experiments.

To further confirm the mixed‐potential‐driven mechanism in CO oxidation, the steady‐state current–potential behaviors for each half‐reaction, i.e., ORR and COOR, were measured on Au NPs and NrGO electrodes, respectively (Figure S4, Supporting Information). The electrodes and electrolytes used to obtain the current–potential curves for COOR and ORR in **Figure** **3** are exactly the same as those used in Figure 2. This was done to enable a direct comparison and to verify the mixed‐potential‐driven mechanism based on the half‐reaction results shown in Figure 3. As the amount of catalyst on the electrode is also identical, the comparison of absolute current values is justified. For ORR, only 0.2 atm O_2_ was introduced into the reactor, while for COOR, only 0.04 atm CO was introduced into the reactor. Figure 3a shows four current–potential curves for ORR and COOR of Au NPs and NrGO, respectively. Note that the unit of the vertical axis is current (A) instead of current density (A cm^−2^) because a mixed potential is determined by the total current independent of electroactive surface area (details provided in Supporting Information). The Au NPs displayed the onset of the anodic oxidation current for COOR (defined at as 10 µA)^[^ ^60^ ^]^ at 0.51 V and rase up to 216 µA at 0.84 V (Figure 3a, red). However, the catalytic current of COOR on NrGO (Figure 3a, pink) was negligible, with almost no current enhancement as the applied potential increased. Thus, we could rule out the COOR occurring on NrGO in the following analysis. The ORR current onset at 0.82 V on the Au NPs electrode and drastically increased to 232 µA at 0.70 V (Figure 3a, green). An onset potential of 0.83 V for ORR on NrGO was observed and its current sharply increased to 193 µA at 0.70 V (Figure 3a, blue).

Here, we address the diffusion of CO and O_2_ in the electrolyte, which is considered to contribute to the observed decline in current under the present experimental conditions. With regards to electrolyte resistance, no current is observed in the absence of PBS (Figure S11, Supporting Information), while a measurable current appears when sufficient PBS is added. This indicates that the ionic resistance of the electrolyte is low under our experimental conditions and is not a dominant factor. The influence of mass transport limitations of CO and O_2_ was evaluated based on the high‐current region of the current–potential curves for COOR and ORR, as shown in Figure 3a. A pronounced plateau in the COOR curve at high currents, clearly visible in both Figure 3a and Figure S9 (Supporting Information), suggests diffusion‐limited behavior. In contrast, no such plateau is observed in the ORR curve, indicating that mass transport does not limit O_2_ reduction under these conditions. These results suggest that CO transport is the rate‐limiting factor for COOR at high currents, whereas ORR is limited by the reaction kinetics rather than mass transport. Even when GDL is employed, CO diffusion remains a critical limiting factor due to the inherently low partial pressure (0.04 atm) and low solubility of CO in water. Accordingly, the gradual decrease in current over time observed in Figure 2c is attributed to progressive deterioration of the GDL, which further impedes CO diffusion to the catalyst surface and exacerbates mass transport limitations.

Using the results in Figure 3a, we predicted the mixed potential of the CO + O_2_ gas mixture measured in the experiment in Figure 2. In Figure 3a, the potential at which the absolute value of the sum of the ORR currents at Au NPs and NrGO equals that of the COOR currents at Au NPs is ≈0.75 V, referred to as the predicted mixed potential ($\phi_{\mathrm{Pre}}^{\mathrm{Mix}}$). This value is remarkably consistent with the $\phi_{\mathrm{Mea}}^{\mathrm{Mix}}$ of 0.75 V shown in Figure 2. Figure 3b illustrates the agreement between predicted values from the current–potential curves and those measured in the short‐circuited experiments. This result strongly supports the occurrence of the mixed‐potential‐driven electrode reactions. The currents through the external circuit ​​were also compared between the results in Figure 2b and Figure 3a, as shown in Figure 3c. The results were consistent within 15%, indicating that the mixed‐potential‐driven CO oxidation allows net current to flow from the NrGO electrode to the Au NPs electrode without the application of an external potential. It should be noted that both the ORR and COOR half‐reactions proceed in a mixed‐potential‐driven mechanism on the Au NPs catalyst as expected from the results in Figure 3a. However, the current due to the local redox half‐reactions could not be measured. The measured short‐circuited current is thus the net current flowing via the external circuit between two electrodes. We further investigated the case where there is no contribution of ORR on the Au NPs catalyst in the H‐cell experiment (Figure S12, see Section S5, Supporting Information for details). That is, we measured the short‐circuited current by introducing only CO on the Au NPs electrode and only O_2_ on the NrGO electrode. As a result, the potential and current values in the H‐cell experiments were in good agreement with the predicted values from the half‐reaction results in Figure 3a.

### 2.3 Measurement of CO_2_ Products and Their Relationship to Current Values

**Figure 4:**
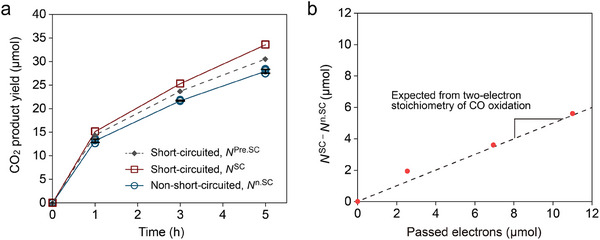
Product yield and electrons estimation. a) CO_2_‐derived product yield as a function of reaction time. Solid lines are products quantified by using UV–vis from short‐circuited (*N* ^SC^) and non‐short‐circuited (*N* ^n.SC^) experiments, respectively. The dotted line is the predicted value from the ratio of reaction currents which are estimated from the current–potential curves of Figure 2a. b) The correlation between product yield difference (*N* ^SC^ − *N* ^n.SC^) and passed electrons. The electrons passed between the two electrodes were estimated by integrating the external short‐circuited current flowing in Figure S15 (Supporting Information). The dotted lines indicate the theoretical value assuming a two‐electron process and are included as visual guides.

To prove the mixed potential‐driven mechanism, the amount of CO_2_ produced in the CO oxidation was quantified using UV–vis absorption spectroscopy.^[^ ^61^, ^62^, ^63^, ^64^ ^]^ Operating the CO oxidation in the aqueous phase traps the CO_2_ in solution by the formation of as CO_2(aq)_, HCO_3_^−^ and CO_3_^2−^.^[^ ^15^, ^65^ ^]^ For accurate CO_2_ quantification, a calibration curve was constructed using standard KHCO_3_ solutions, with the pH maintained by PBS buffer (the same as the reaction electrolyte). This curve correlated the UV–vis absorbance of HCO_3_^−^ (vertical axis) with the total concentration of CO_2_‐derived species (horizontal axis), as shown in Figure S15 (Supporting Information). Four key factors were considered: i) an acid–base equilibrium should be considered among CO_2(aq)_, HCO_3_^−^, and CO_3_^2−^, described by CO_2(aq)_ + H_2_O ⇆ HCO_3_^−^ + H^+^ ⇆ CO_3_^2−^ + 2H^+^; ii) Because the species composition ratios are strongly dependent on pH, the pH was standardized to 7.2 in the calibration and CO oxidation experiments; iii) At pH 7.2, the concentration of HCO_3_^−^ is dominant (87.6%) and most representative of total dissolved CO_2_‐derived species (Figure S14, Supporting Information); iv) the amount of CO_2_‐derived species from CO oxidation was estimated from the HCO_3_^−^ peak intensity in UV–vis spectroscopy based on the prepared calibration curve.^[^ ^61^, ^64^ ^]^ In control experiments under Ar or O_2_ atmosphere, HCO_3_^−^ is not detected by UV–vis (Figure S18, Supporting Information). To further verify the capture of CO_2_ in the solution, we conducted an additional short‐circuited experiment at pH 11.9, observing a decrease in pH to 10.3 after the reaction (Figure S19, Supporting Information). **Figure** **4**a shows the products in the short‐circuited and non‐short‐circuited experiments with the flow of CO+O_2_ gas mixture at 1, 3, and 5 h. The observation of CO_2_ products in the non‐short‐circuited experiments can be explained by the local mixed‐potential‐driven reactions on Au NPs, as previously discussed in the results of Figure 3. The decrease of product yield rate was consistent with the observed drop in short‐circuited current over time (Figure 2c; Figure S6, Supporting Information).

The relative contributions of the local mixed‐potential‐driven reaction at the Au electrode and the electrochemical coupling between NrGO and Au were evaluated by comparing the trends in product yields and estimated currents at mixed potentials. As shown in Figure 4a, the difference in CO_2_‐derived product amounts between the non‐short‐circuited (*N* ^n.SC^) and short‐circuited (*N* ^SC^) conditions consistently remained ≈10% throughout the entire reaction time. Notably, this 10:1 ratio between the reaction contributions at the Au electrode and those at the NrGO–Au pair was also reflected in the estimated current values at mixed potential from Figure S16 (Supporting Information) and Figure 3a (160 µA for the Au electrode alone and 176 µA for the NrGO–Au pair), respectively, indicating a stable and reproducible division of electrochemical activity across both measurements (see Section S7, Supporting Information). Assuming a linear relationship between current and product formation, a predicted value under short‐circuited conditions was estimated as *N* ^Pre.SC^ = *N* ^n.SC^  + 0.1 × *N* ^n.SC^, shown as a dotted line in Figure 4a. The overall agreement between *N* ^SC^ and *N* ^Pre.SC^ supports the validity of this model in capturing the observed product trend. The slight discrepancy between the measured and the predicted values may be attributed to the accuracy of the current values in the current–potential measurements. In Figure 3b, the mixed potential was consistent between the predicted and measured values, but there was a minor difference in the current values. In addition, there may be a contribution of CO_2_ production due to thermal reactions, but we consider that the contribution in aqueous systems is small.

In addition, the relationship between the amount of CO_2_‐derived product and the number of electrons flowing by the two‐electron reaction provides evidence for the mixed‐potential‐driven mechanism by Au NPs and NrGO. The vertical axis of Figure 4b is the difference between the amount of CO_2_‐derived products in the short‐circuited and non‐short‐circuited conditions (*N* ^SC^ − *N* ^n.SC^), while the horizontal axis of Figure 4b is the integral of the number of electrons calculated from the short‐circuited current measured in Figure S17 (Supporting Information). Remarkably, all data points in Figure 4b fall precisely on the theoretical line corresponding to the two‐electron reaction, as described in Equations (1)–(4). This observation can only be explained by the mixed‐potential‐driven mechanism, ruling out alternative explanations. Additionally, the measured mixed potential decreases by 54 mV per pH unit, which closely approximates the 59 mV per pH unit, indicating a 1:1 proton‐to‐electron stoichiometry (Figure S20, Supporting Information).^[^ ^66^, ^67^ ^]^ Above correlation is in exact agreement with the expected two‐electron stoichiometry for mixed‐potential‐driven CO oxidation.

The most significant contribution of this study is the clear experimental demonstration of a mixed‐potential‐driven mechanism analogous to corrosion. Notably, this system exhibits electrochemical coupling between the two half‐reactions even though the NrGO and Au electrodes are physically separated. In typical corrosion processes, anodic and cathodic reaction sites are located in close proximity; however, in this study, the two electrodes were separated by a distance of ≈1 cm. In the experiment shown in Figure 2, PBS was added to serve as the electrolyte. Almost no reaction current was detected when pure water was used, indicating the necessity of ionic conductivity. Upon the addition of sufficient electrolyte, the mixed‐potential‐driven catalytic reaction proceeded efficiently, and the resistance associated with ionic transport remained negligible.

This study was conducted under liquid electrolyte conditions rather than humidified gas phase conditions. Nevertheless, as discussed in the Introduction, it is plausible that adsorbed water on the support surface facilitates ionic species transport. In particular, the acid‐base function of metal oxide surfaces could act as an electrolyte, and the presence of water could improve the electrolyte function. For example, in the study of the Au/TiO_2_ (110) model catalyst by Fujitani et al., negligible CO oxidation activity was observed at 300 K in the absence of water vapor.^[^ ^21^ ^]^ However, when water vapor was introduced at partial pressures ranging from 0 to 0.5 Torr at the same temperature, a significant increase in catalytic activity was observed. Moreover, the activity was found to correlate with the perimeter of the Au particles, i.e., the interface between the Au particles and the TiO_2_ surface. It is also notable that the desorption temperature of water adsorbed on TiO_2_ (110) is ≈370 K.^[^ ^30^ ^]^ Thus, at the reaction temperature of 300 K, the TiO_2_ surface is likely saturated with water‐derived adsorbed species. This suggests that the adsorbed water may function as an ion‐conducting medium, enabling electrochemical reactions at the perimeter between Au and TiO_2_. This interpretation is consistent with previous findings showing that catalytic activity is localized at the Au/TiO_2_ perimeter under such conditions, with the activity being proportional to the perimeter length. The Au surface remains dry while the TiO_2_ (110) surface remains wet, thus forming an electrochemical three‐phase boundary at the perimeter of Au particles where the reaction is presumed to take place. However, the present study does not provide conclusive evidence that the mixed‐potential‐driven mechanism operates under humidified conditions. For example, we cannot exclude the possibility that OOH is formed by thermal activation of O_2_ and H_2_O,^[^ ^20^, ^59^, ^68^ ^]^ and further investigations are required to elucidate the underlying mechanism.

## 3 Conclusion

This study, employing a model electrode system, provides clear evidence that room‐temperature CO oxidation over Au catalysts in the aqueous electrolytes proceeds via a mixed‐potential‐driven mechanism—namely, the electrochemical coupling of COOR and ORR. In the experiments, two spatially separated catalyst electrodes were exposed to a mixed atmosphere of CO and O_2_. Upon electrically short‐circuiting the electrodes, a reaction current was observed without the application of an external potential, with its magnitude dependent on the intrinsic catalytic activity. To probe the viability of the mixed‐potential‐driven mechanism, Au nanoparticles (Au NPs) and nitrogen‐doped reduced graphene oxide (NrGO) electrodes were selected as model catalysts. The experimentally measured mixed potential, short‐circuited current, current–potential characteristics for the individual half‐reactions (COOR and ORR), and the amount of CO_2_‐derived products were all in quantitative agreement with the predictions of the mixed‐potential model. Given that literature‐reported turnover frequencies (TOFs) for CO oxidation under humidified gas‐phase and liquid‐electrolyte conditions are comparable, we propose that the mixed‐potential‐driven mechanism may also operate in humidified environments. Although the mechanism of Au‐catalyzed CO oxidation has been extensively investigated and the promotional role of water is well documented, our findings provide a more comprehensive mechanistic explanation based on electrochemical coupling. While we do not entirely rule out a thermal (non‐electrochemical) contribution to CO oxidation, our results strongly support the dominant role of the electrochemical pathway in the observed reactivity.

## Conflict of Interest

The authors declare no conflict of interest.

## Supporting information



Supporting Information

## Data Availability

The data that support the findings of this study are available from the corresponding author upon reasonable request.
