# Supplementary material for: Mixed‐Potential‐Driven Catalysis: An Electrochemical Mechanism for Room‐Temperature CO Oxidation on Gold Catalysts
Source: Adv Sci (Weinh). 2025 Jun 25;12(35):e05994. doi: 10.1002/advs.202505994 (PMC12462927; doi:10.1002/advs.202505994)
Supplement: Supplementary file 1 — Supporting Information [file ADVS-12-e05994-s001.docx]

Supporting Information for

**Mixed-Potential-Driven Catalysis: An Electrochemical Mechanism for Room-Temperature CO Oxidation on Gold Catalysts**

Mo Yan,^[a]+^ Asif Muhammad,^[b]+^ Ravi Singh,^[b]^ Kotaro Takeyasu,*^[c,d,e,f]^ Junji Nakamura*^[a]^

Corresponding author: [takeyasu@cat.hokudai.ac.jp](mailto:takeyasu@cat.hokudai.ac.jp); [nakamura.junji.700@m.kyushu-u.ac.jp](mailto:nakamura.junji.700@m.kyushu-u.ac.jp)

**The** **Supporting Information includes:**

Materials and Methods

Supplementary Text

Figs. S1 to S20

Tables S1 to S5

References

**Table of Contents**

[Materials and Methods 3](#_Toc198370534)

[Chemicals and materials 3](#_Toc198370535)

[Preparation of gas diffusion electrodes 3](#_Toc198370536)

[Characterization of deposited Au NPs and NrGO 3](#_Toc198370537)

[Measurements of short-circuited currents and measured mixed potentials 5](#_Toc198370538)

[Measurements of current–potential curves 6](#_Toc198370539)

[The unit of the vertical axis of current–potential curves and short-circuited currents 6](#_Toc198370540)

[Product detection by UV–vis spectroscopy 7](#_Toc198370541)

[Supplementary Text 7](#_Toc198370542)

[S1. Comparison of the kinetic behavior of CO oxidation in the gas phase (with and without H_2_O vapor) and in the liquid phase 7](#_Toc198370543)

[S2. Long-time short-circuit experiments 9](#_Toc198370544)

[S3. Control experiments using an unmodified carbon paper coupled with NrGO or Au NPs 9](#_Toc198370545)

[S3. Impact of mass transport 11](#_Toc198370546)

[S5. Short-circuited experiments in the H-cell 13](#_Toc198370547)

[S6. Calibration processes 15](#_Toc198370548)

[S7. Product yield estimation for non-short-circuited and short-circuited conditions 17](#_Toc198370549)

[S8. Measured mixed potential versus pH 21](#_Toc198370550)

[References 22](#_Toc198370551)

Materials and Methods

Chemicals and materials

The solutions were freshly prepared using ultrapure water (Millipore, 18.2 MΩ, TOC < 4 ppb). 0.25 M phosphate buffer solution (PBS) was purchased from Hayashi Pure Chemical Ind., Ltd. Potassium hydroxide (KOH), sodium bicarbonate (NaHCO_3_), and perchloric acid (HClO_4_) were purchased from Sigma-Aldrich. Solutions of different pH were prepared by dissolving the appropriate amount of KOH in 0.1 M HClO_4_. Carbon paper was purchased from MFC Technology Co., Ltd. Nafion® (5 wt% in lower aliphatic alcohols and water, contains 15-20% water) was purchased from Sigma-Aldrich. All chemicals were used as received without any further purification.

Nitrogen-doped reduced graphene oxide (NrGO) was synthesized similar to a previous study ^[1]^ as follows. The Hummer’s method was employed in the synthesis of graphene oxide (GO) from graphite powder, followed by the fabrication of NrGO through a thermal annealing process. Specifically, 0.2 g of GO was measured and placed within a quartz glass boat. Subsequently, the quartz glass boat was positioned at the center of a tubular furnace. The temperature was gradually increased to 500 °C with a N_2_ flow (5 cm^3^ min^−1^). During the temperature elevation from 500 to 750 °C, NH_3_ (5 cm^3^ min^−1^) was flowed. Holding time was 3 hours. The temperature ramping rate was 5 °C min^−1^ throughout the synthesis. Following the 3-hour nitrogen doping period with NH_3_, the furnace was systematically cooled, resulting in the successful attainment of NrGO.

Preparation of gas diffusion electrodes

The experimental procedure for depositing NrGO powder onto the electrode was as follows. (1) A catalyst ink was made by adding 5 mg of the catalyst powder into 1 mL of a 3:2 mixture of isopropyl alcohol (IPA) and distilled water, with 40 μL Nafion® solution (5 wt%). (2) The ink was sonicated for 40 min. (3) Then 80 μL of the ink was dropped in 20 μL droplet increments onto the surface of the carbon paper over a hotplate set at 80 °C. This loading required the deposition of ink layers in sequence after the previous layer of air drying. Catalyst plates were then left to dry at room temperature for 12 h. (4) The prepared catalyst loading on carbon paper was fixed by electrode holders to be the gas diffusion electrode (GDE). The Au NPs were deposited on the carbon paper using a sputtering apparatus. This deposition method was selected for its simplicity and effectiveness in producing well-distributed nanoscale particles.^[2,3]^ The sputter conditions were as follows: working distance, 50 mm; sputtering time, 15 min; applied voltage, 1000 V; and discharge current, 30 mA. Then, the Au NP–loaded carbon paper was mounted in electrode holders and used as the gas diffusion electrode (GDE). For all experiments in this study, the catalyst films covered an approximately geometric area of 1 cm^2^, unless otherwise specified.

Characterization of deposited Au NPs and NrGO

The scanning electron microscopy (SEM) images were measured by SEM S-4800 (Hitachi Co. Ltd, Tokyo, Japan) at an accelerating voltage of 10 kV. **Figure S1** shows the representative SEM images of deposited Au NPs before and after reaction.


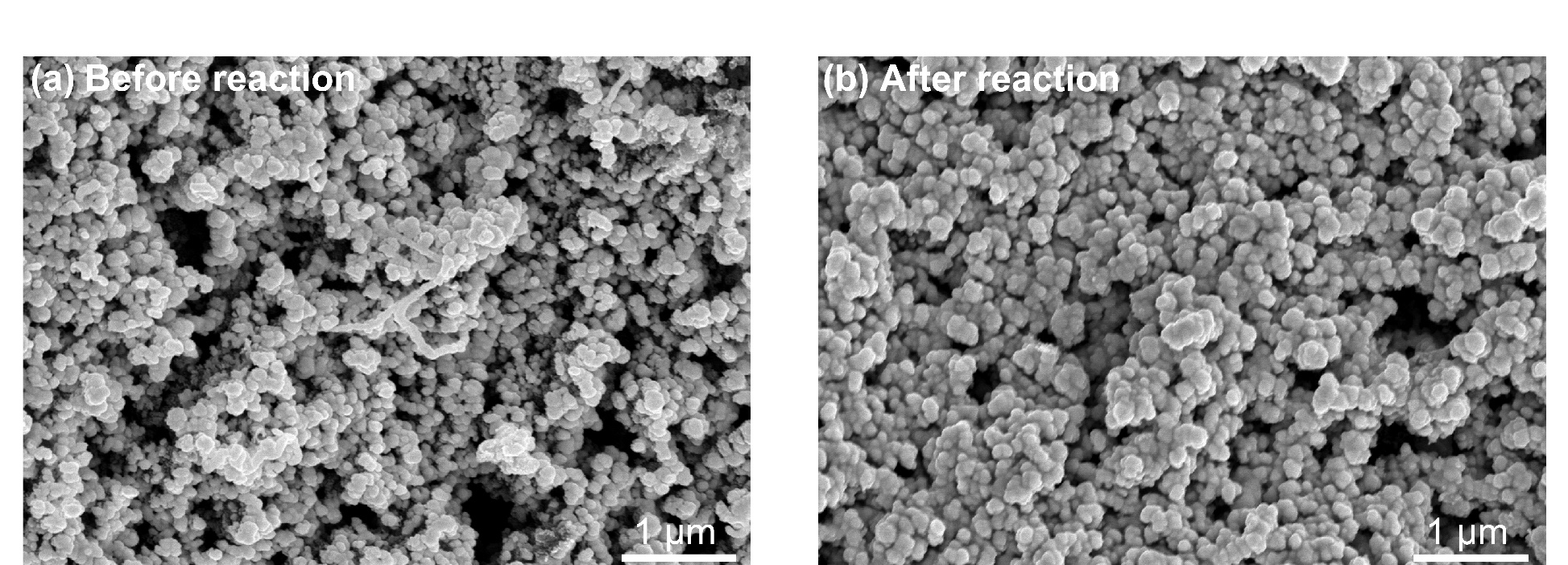


**Fig. S1**. Representative SEM images of deposited Au NPs on carbon paper (a) before and (b) after short-circuited reaction in Fig. 2.

X-ray photoelectron spectroscopy (XPS) was conducted on JPS 9010 TR, JEOL Ltd., with an X-ray source of MgKα, 1253.6 eV, and pass energy of 20 eV. The binding energy of the XPS spectrum was calibrated based on the peak energy positions of the standard samples. The XPS data for deposited Au NPs before and after reaction was shown in **Fig. S2**.


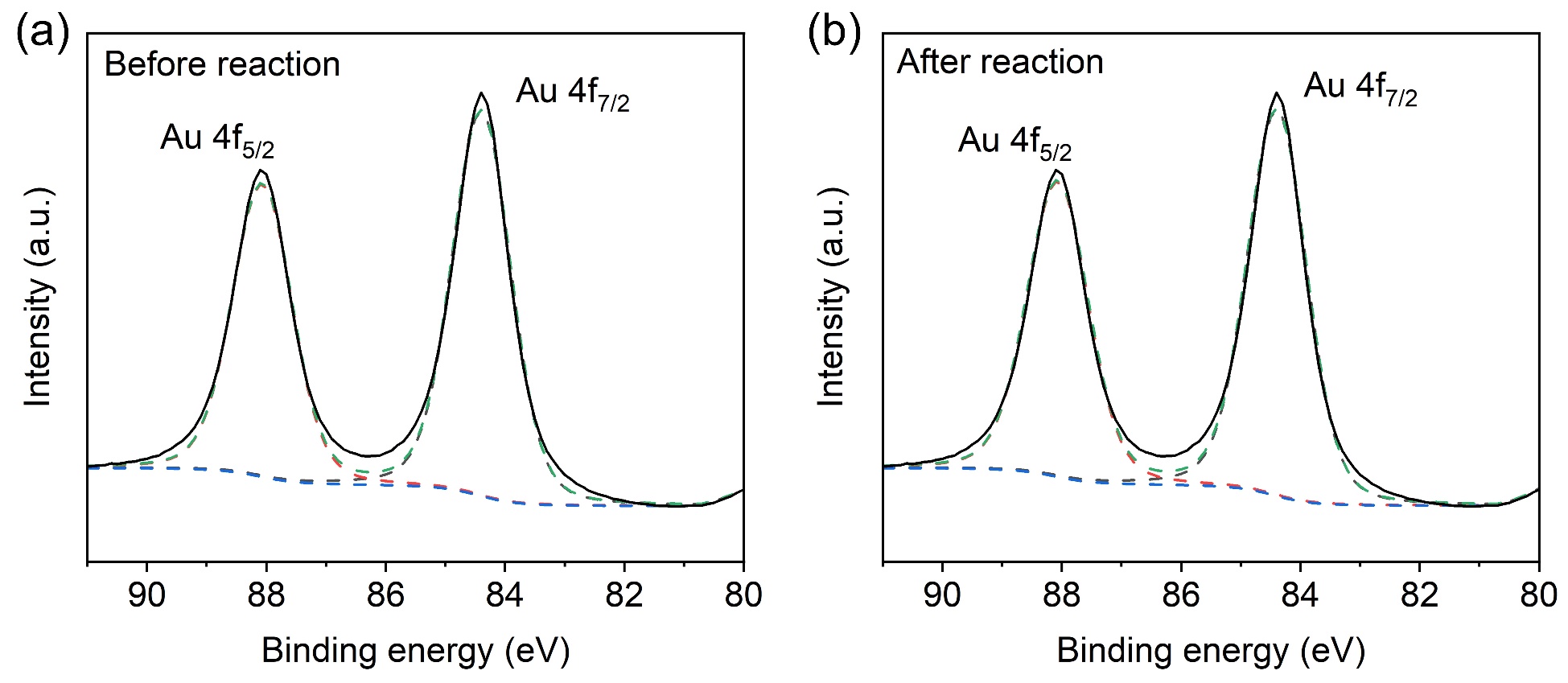


**Fig. S2**. XPS data of Au 4f region for deposited Au NPs (a) before and (b) after short-circuited experiments in Fig. 2.

The atomic percentages of C, N, and O were obtained after background subtraction using Shirley-type background subtraction performed using SPECSURF Analysis software. After that, to reveal the configuration of nitrogen, carbon, and oxygen contained in the material, CasaXPS software was used. In the fitting using CasaXPS, the binding energy was fixed according to the references. The XPS data for NrGO was shown in **Fig. S3**. The atomic percentages of C, doped-N, and O were 92.84%, 3.98%, and 3.18%, respectively. Among which, the different species of N and C were summarized in **Tables S1** and **S2**, respectively.


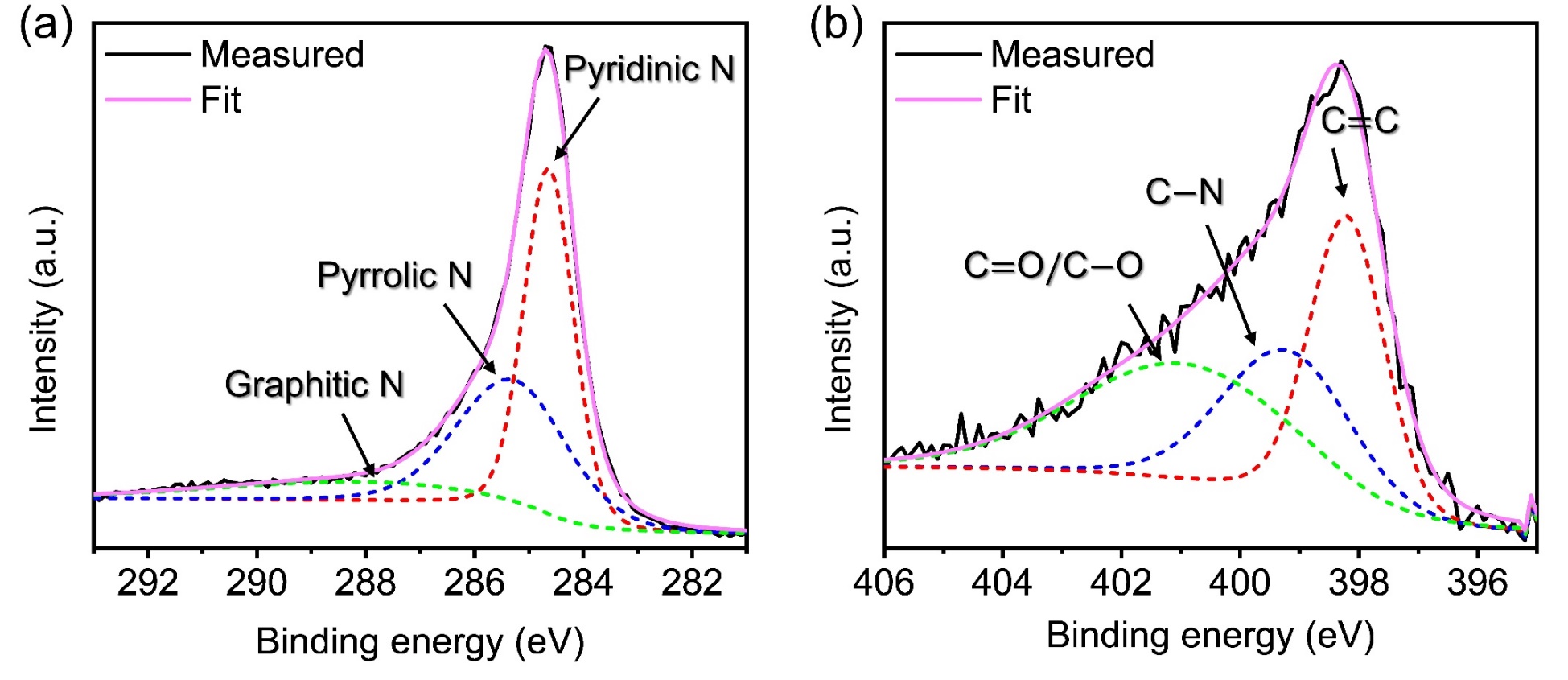


**Fig. S3**. XPS data of N1s and C1s region for NrGO.

**Table S1**. Atomic percentages of different N species in NrGO

| N species | Peak energy (eV) | Atomic % |
| --- | --- | --- |
| Pyridinic | 398.2 | 1.32 |
| Pyrrolic | 399.2 | 1.14 |
| Graphitic | 400.9 | 1.52 |

**Table S2**. Atomic percentages of different C species in NrGO

| C species | Peak energy (eV) | Atomic % |
| --- | --- | --- |
| C=C | 284.2 | 43.1 |
| C–N | 285.3 | 36.1 |
| C=O | 288.0 | 13.7 |

Measurements of short-circuited currents and measured mixed potentials

The deposited Au NPs over carbon paper and NrGO over carbon paper were employed as the macroscopic GDEs. The two hanging-strip GDEs were inserted into the compartment of a single cell, which was filled with the reaction solution (0.25 M PBS, pH 7.2) with a volume of 15 mL. As shown in **Fig. 2a**, the desired gas was continually flowed into the single cell at a controlled flow rate using a series of mass-flow controllers. The composition of Ar cylinder and O­_2_ cylinder is ultrahigh-purity (99.99%) Ar and O_2_, respectively. The composition of CO/Ar cylinder is 5% CO balanced by 95% Ar. The partial pressures of CO and O­_2_ are determined by their respective flow rates.

Before the experiment, the reaction solution was sparged for >30 min with Ar gas at a flow rate 50 cm^3^ min^−1^ to ensure removal of the residual O_2_. Then, the reaction was initiated by introducing one of the following feeds: (1) ultrahigh-purity O_2_ at a flow rate 10 cm^3^ min^−1^ and Ar gas at a flow rate 40 cm^3^ min^−1^; (2) 5% CO balanced by Ar at a flow rate 40 cm^3^ min^−1^ and Ar gas at a flow rate 10 cm^3^ min^−1^; (3) 5% CO balanced by Ar at a flow rate 40 cm^3^ min^−1^, and ultrahigh-purity O_2_ at a flow rate 10 cm^3^ min^−1^. The partial pressures of CO and O_2_ were 0.04 atm and 0.2 atm, respectively, across all the feeding conditions.

Shorting the two electrodes via an external circuit was sufficient to drive the overall CO oxidation. During the reaction, the short-circuited current between the Au NPs and NrGO electrodes was monitored using a potentiostat. The electrodes were connected as follows: two working electrode clips were connected to the Au NPs electrode, counter and reference clips were connected to the NrGO electrode. The mixed potential was measured by a second potentiostat in the open-circuit potential measurement mode, with two working electrode clips connected to the short-circuited Au NPs and NrGO electrodes, and the counter and reference clips connected to a leakless Ag/AgCl reference electrode. All experiments were conducted at room temperature (25 ± 1 °C).

Measurements of current–potential curves

The steady-state current–potential curves were collected by using chronoamperometry method (Metrohm Autolab potentiostat-galvanostat, PGSTAT302N) in a single cell containing the reaction solution (0.25 M PBS, pH 7.2, 15 mL). A platinum wire served as the counter electrode and a leakless Ag/AgCl electrode was used as the reference electrode. Electrode potentials were converted to the RHE (*E*_RHE_) scale using the equation *E*_RHE_ = *E*_Ag/AgCl_ + 0.197 V + 0.059 × pH. Before the experiments, the working electrode underwent a surface cleaning step by performing cyclic voltammetry (50 mV s^−1^) for 35 cycles (–0.2 to 1.2 V *vs* RHE) under an Ar flow of 50 cm^3^ min^−1^. The setup is shown in **Fig. S4**. For the electrochemical CO oxidation half-reaction (COOR), 4% CO balanced by Ar was continuously flowed to the working compartment at a rate of 50 cm^3^ min^−1^. For the electrochemical O_2_ reduction half-reaction (ORR), 20% O_2_ balanced by Ar was continually flowed to the working compartment at a rate of 50 cm^3^ min^−1^. Potentials were not corrected for uncompensated Ohmic loss (*iR*^u^).

Chronoamperometry measurements were performed at 10 mV intervals, with each potential held for 20 seconds. To eliminate the effects of capacitive charging, the initial two seconds of data at each potential step were discarded. The average of the remaining current values was used to determine the steady-state current for each potential. The catalyst films used covered an approximate geometric area of 1 cm², unless otherwise specified. All experiments were conducted at room temperature (25 ± 1 °C).


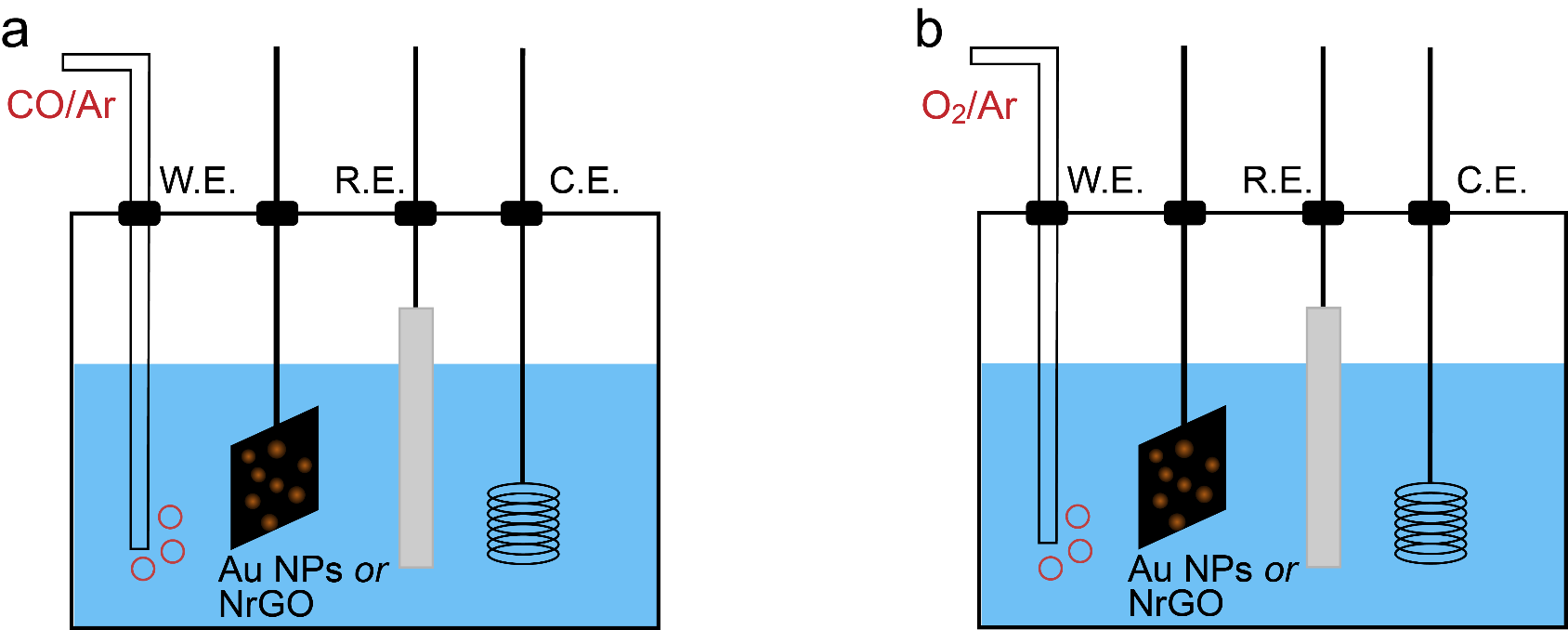


**Fig. S4.** Experimental configurations utilized for measuring current–potential curves of (a) COOR on Au NPs and NrGO and (b) ORR on Au NPs and NrGO. For the COOR, 4% CO balanced by Ar was continuously flowed to the working compartment at a rate of 50 cm^3^ min^−1^. For the ORR, 20% O_2_ balanced by Ar was continually flowed to the working compartment at a rate of 50 cm^3^ min^−1^.

The unit of the vertical axis of current–potential curves and short-circuited currents

The mixed potential is the point at which two or more distinct half-reactions combine to establish a potential with a net zero external current. Notably, the mixed potential condition corresponds to zero net current (A), not current density (A cm^−2^). It has been observed that mixed potentials, as well as current–potential curves, are highly dependent on the electroactive area of the electrode.^[4]^ Therefore, using area-normalized current, i.e., current density (A cm^−2^), as the vertical axis is unsuitable for our study.

For example, consider a metallic nanoparticle (NP) immobilized on a support. The support has a significantly higher electroactive surface area than the metallic NP. If the cathodic half-reaction exhibits very slow kinetics on the support (i.e., a small exchange current density), while the anodic half-reaction demonstrates favorable kinetics on the metallic NP, the sluggish kinetics of the cathodic half-reaction can be compensated by the larger electroactive area of the support. This compensation results in a current comparable to that of the anodic half-reaction on the metallic NP.^[4]^

Product detection by UV–vis spectroscopy

Our experiments were conducted in 0.25 M PBS at pH 7.2, where CO_2_ was captured in solution as carbonic acid and bicarbonate. The bicarbonate concentration in the solution was determined by UV absorbance in the range of 195 to 215 nm.^[5–7]^ Thus, using UV absorbance as a proxy for bicarbonate concentration provides confirmatory evidence of dissolved CO₂ from CO oxidation. The UV–vis spectra were recorded with a Jason V-630 BIO spectrophotometer using a 1 cm path length cuvette at a scan rate of 40 nm min^−1^.

Supplementary Text

S1. Comparison of the kinetic behavior of CO oxidation in the gas phase (with and without H_2_O vapor) and in the liquid phase

Turnover frequency (TOF) data compiled from literature sources reveal a pronounced enhancement of CO oxidation activity over supported Au catalysts in the presence of water vapor and liquid water (**Fig. S5**). In the gas phase, the introduction of water vapor significantly increases TOFs across a range of supports, as shown in **Fig. S5a** and **Table S3**. Carbon-supported Au catalysts that were inactive in the vapor-phase oxidation of CO demonstrated significant activity in the liquid phase, as shown in **Fig. S5b and Table S4**. This sharp contrast underscores the unique promotional role of interfacial water.


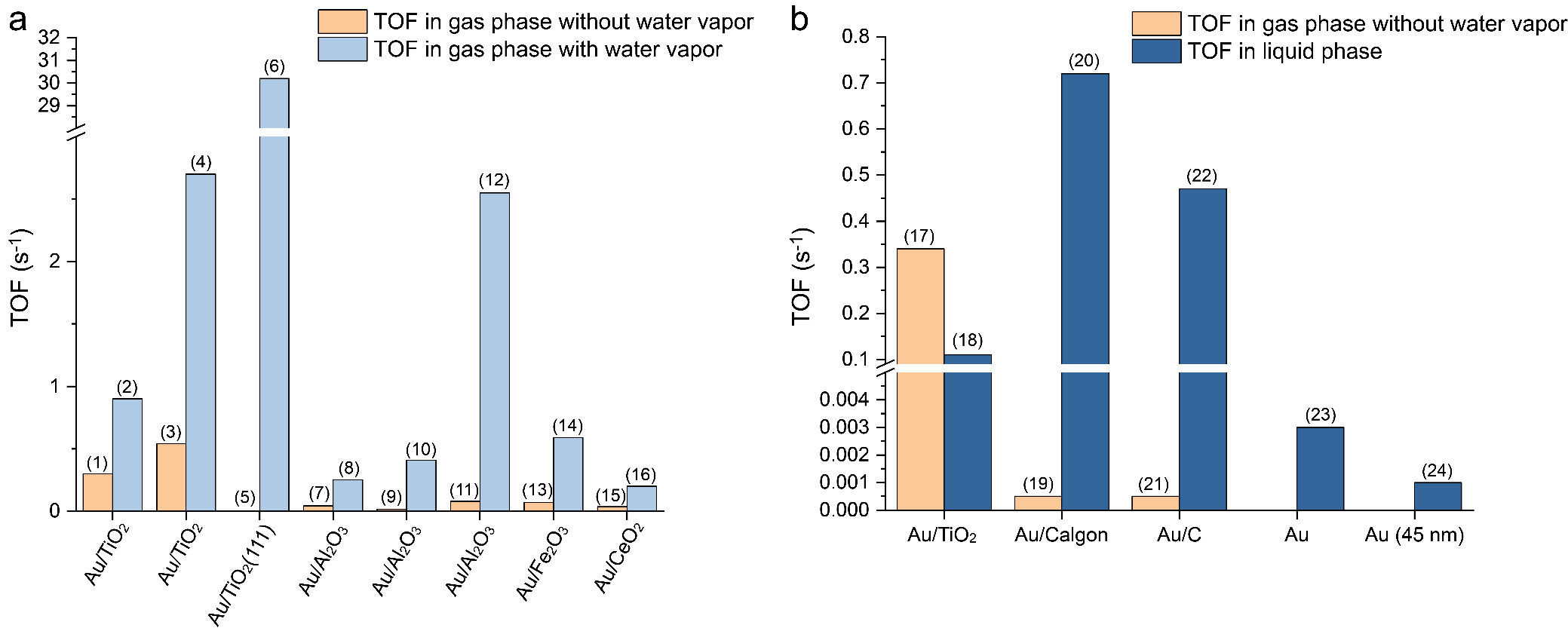


**Fig. S5** TOF of CO oxidation over supported Au catalysts in different reaction conditions: (a) gas phase with and without water vapor, and (b) gas phase versus liquid phase.

**Table S3**. Comparison of CO oxidation activities (TOF) in the gas phase with and without water

| **Catalysts** | **Au size (nm)** | $\text{T }\left( \text{K} \right)$ | $\text{p}_{\text{O}_{\text{2}}}\text{(kPa)}$ | | $\text{p}_{\text{CO}}\text{(kPa)}$ | | $\text{p}_{\mathbf{H}_{\mathbf{2}}\mathbf{O}}\text{ }\text{(Pa)}$ | | **TOF (s^−1^)** | | **No.** | |
| --- | --- | --- | --- | --- | --- | --- | --- | --- | --- | --- | --- | --- |
| Au/TiO_2_ | 2.9 | 293 | 20.1 | | 1 | | 0 | | 0.3 | | (1)^[8]^ | |
| Au/TiO_2_ | 2.9 | 293 | 20.1 | | 1 | | 800 | | 0.9 | | (2)^[8]^ | |
| Au/TiO_2_ | 3.3 | 288 | 2 | | 5 | | 0 | | 0.54 | | (3)^[9]^ | |
| Au/TiO_2_ | 3.3 | 288 | 2 | | 5 | | 500 | | 2.70 | | (4)^[9]^ | |
| Au/TiO_2_(110) | - | 300 | 83.3 | | 3.3 | | 0 | | 0 | | (5)^[10]^ | |
| Au/TiO_2_(110) | - | 300 | 83.3 | | 3.3 | | 13.33 | | 30.2 | | (6)^[10]^ | |
| Au/Al_2_O_3_ | 1.2 | 295 | 2.4 | | 2.4 | | 0 | | 0.042 | | (7)^[11]^ | |
| Au/Al_2_O_3_ | 1.2 | 296 | 2.4 | | 2.4 | | 218.9 | | 0.25 | | (8)^[11]^ | |
| Au/Al_2_O_3_ | 1.2 | 298 | 3.0 | | 3.0 | | 0 | | 0.016 | | (9)^[12]^ | |
| Au/Al_2_O_3_ | 1.2 | 298 | 3.0 | | 3.0 | | 810.6 | | 0.41 | | (10)^[12]^ | |
| Au/Al_2_O_3_ | 3.5 | 288 | 2 | | 5 | | 0 | | 0.08 | | (11)^[9]^ | |
| Au/Al_2_O_3_ | 3.5 | 288 | 2 | | 5 | | 500 | | 2.55 | | (12)^[9]^ | |
| Au/Fe_2_O_3_ | 3.6 | 288 | 2 | | 5 | | 0 | | 0.07 | | (13)^[9]^ | |
| Au/Fe_2_O_3_ | 3.6 | 288 | 2 | | 5 | | 500 | | 0.59 | | (14)^[9]^ | |
| Au/CeO_2_ | 5 | 298 | 1 | | 1 | | 2026.5 | | 0.036 | | (15)^[13]^ | |
| Au/CeO_2_ | 5 | 298 | 1 | 1 | | 2026.5 | | 0.2 | | (16)^[13]^ | |  |

**Table S4**. Comparison of CO oxidation activities (TOF) in the gas phase and in the liquid phase *^a^*

| **Catalysts** | **Au size (nm)** | $\text{T }\left( \text{K} \right)$ | $\text{p}_{\text{O}_{\text{2}}}$**or** ${\text{[}\text{C}\text{]}}_{\text{O}_{\text{2}}}$***^b^*** | $\text{p}_{\text{CO}}$ **or** ${\text{[}\text{C}\text{]}}_{\text{O}_{\text{2}}}$ | **Gas or liquid phase** | **TOF (s^−1^)** | **No.** |
| --- | --- | --- | --- | --- | --- | --- | --- |
| Au/TiO_2_ | 3.7 | 293 | 2 kPa | 2 kPa | Gas | 0.34 | (17)^[14]^ |
| Au/TiO_2_ | 3.7 | 300 | 0.65 mM | 0.8 mM | Liquid | 0.11 | (18)^[14]^ |
| Au/Calgon | 5 | 293 | 1.5 | 3 | Gas | < 0.0005 | (19)^[14]^ |
| Au/Calgon | 5 | 300 | 0.65 mM | 0.8 mM | Liquid | 0.72 | (20)^[14]^ |
| Au/C | 10.5 | 293 | 1.5 | 3 | Gas | < 0.0005 | (21)^[14]^ |
| Au/C | 10.5 | 300 | 0.65 mM | 0.8 mM | Liquid | 0.47 | (22)^[14]^ |
| Au powder | - | 300 | 0.65 mM | 0.8 mM | Liquid | 0.003 | (23)^[15]^ |
| Au powder | 45 | 300 | 0.65 mM | 0.8 mM | Liquid | 0.001 | (24)^[15]^ |

*^a^* The pH value for reaction in the liquid phase is 7.

*^b^* Utilizing a partial pressure of 1 atm CO in equilibrium with the aqueous phase gives a dissolved CO concentration of ~0.8 mM, which corresponds to a CO partial pressure of 0.02 atm in gas phase.

**Table S5** summarizes CO oxidation rates at 300 K and 1 bar total pressure of a CO:O_2_ ratio of 1:1 in a membrane reactor.^[16]^ In the absence of water, the CO_2_ production rate is minimal (3 μmol g_Au_^−1^ min^−1^). Introducing 30 mbar of water vapor into the gas stream increases the activity tenfold (30 μmol g_Au_^−1^ min^−1^), highlighting the promotional effect of humidity. Contracting the Au membrane with liquid water further raises the rate to 47 μmol g_Au_^−1^ min^−1^.

**Table S5**. Comparison of CO oxidation activities in the gas phase, liquid phase, and fluidic phase over Au nanotube in a membrane reactor ^[16]^

| **Gas chamber *^a^*** | **Membrane** | **Liquid chamber *^b^*** | **CO_2_ rate *^c^*** |
| --- | --- | --- | --- |
| CO:O_2_ | Au | None | 3 |
| CO:O_2_ H_2_O (30 mbar) | Au | None | 30 |
| CO:O_2_ | Au | H_2_O | 47 |

*^a^* Total gas flow rate = 100 cm^3^ min^−1^; CO:O_2_ ratio = 1:1.

*^b^* Liquid flow rate = 2 cm^3^ min^−1^.

*^b^* In units of mmol of CO_2_ per gram of Au per minute.

S2. Long-time short-circuit experiments

A long-term experiment was conducted to observe the trend and plateau of the short-circuited current and mixed potential under the flow of CO and CO+O_2_, as shown in **Fig. S6**. CO (0.04 atm) was continuously introduced into the single cell for 24 hours, and then, CO (0.04 atm) was switched to CO (0.04 atm) + O_2_ (0.2 atm) gas for 35 hours.


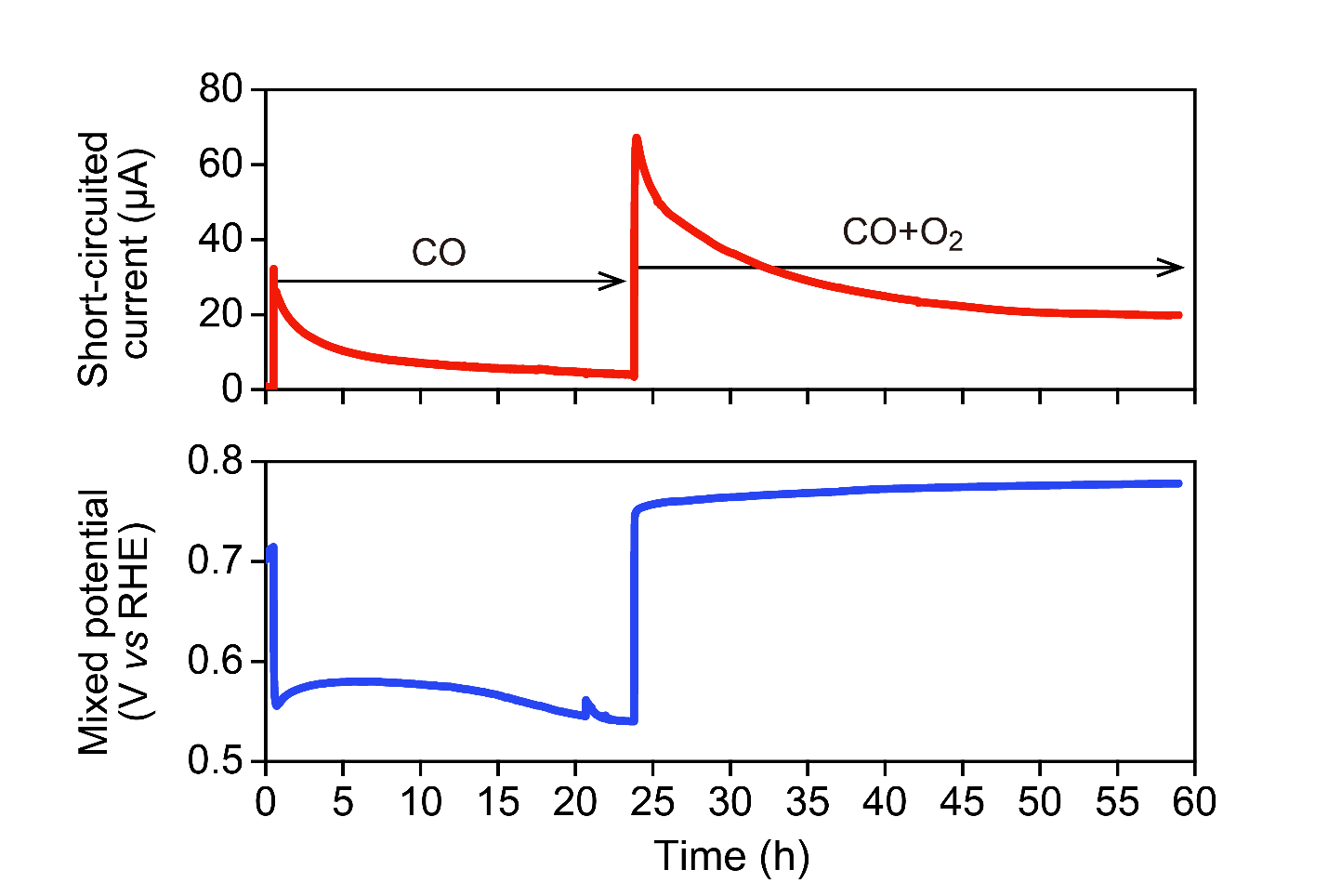


**Fig. S6**. Long-term measurements of the short-circuited current and mixed potential were conducted. To obtain the steady-state current, CO (0.04 atm) was continuously introduced into the single cell for 24 hours, after which the feed was switched to a mixture of gas CO (0.04 atm) + O_2_ (0.2 atm) for 35 hours. Reaction conditions: 0.25 M PBS (15 mL, pH 7.2); 25 ± 1 °C; total flow rate: 50 cm^–3^ min^–1^.

S3. Control experiments using an unmodified carbon paper coupled with NrGO or Au NPs

In the flowing control short-circuited experiments, the reaction conditions were as follows: 0.25 M PBS (15 mL, pH 7.2), 25 °C, with the feed periodically switching between Ar, CO (0.04 atm), O_2_ (0.2 atm), and a mixture of CO (0.04 atm) + O_2_ (0.2 atm). The total flow rate was maintained at 50 cm^3^ min^−1^.

1. In the absence of Au NPs but in the presence of NrGO

In a control experiment where Au NPs on carbon paper were replaced with unmodified carbon paper (as shown in **Fig. S7**), negligible short-circuited currents were observed under Ar and CO feeds. Intriguingly, the introduction of O_2_ resulted in measurable short-circuited currents, approximately 6 to 9 μA, and a positive shift of mixed potential, indicating an electrochemical oxidation half-reaction on the carbon paper coupled with the ORR on NrGO side. This observation aligns with reports in the literature that carbon paper can undergo electrochemical self-oxidation.^[17,18]^

In other words, the oxidation half-reaction on the Au NPs (**Fig. 2**) and unmodified carbon paper (**Fig. S7**) is driven by electrochemical ORR on NrGO. Importantly, almost no short-circuited current was detected under CO feed and the short-circuited current did not change upon the addition of O_2_ to CO. This highlights the critical role of Au NPs for CO oxidation.


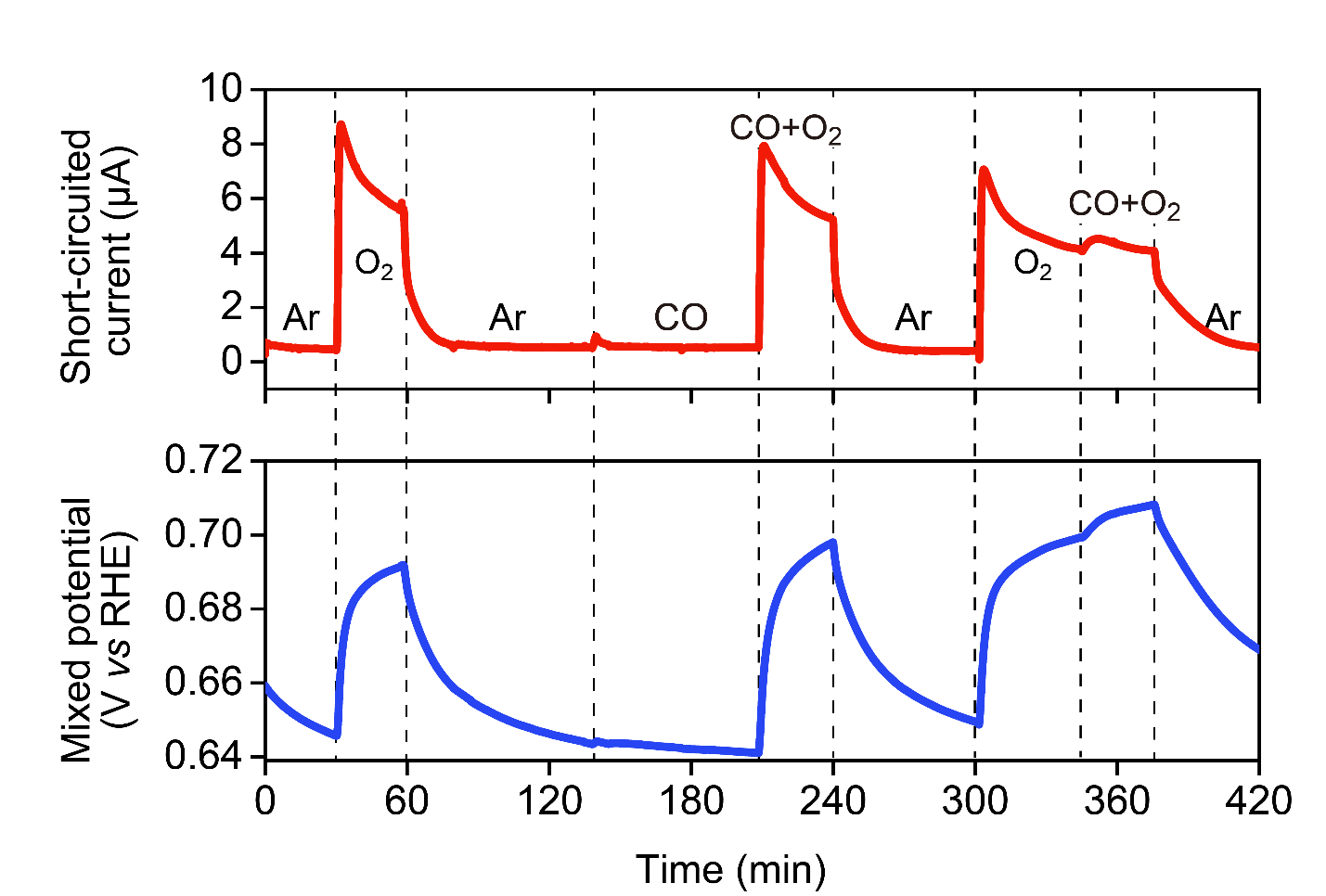


**Fig. S7**. Short-circuit current and mixed potential as a function of time in a control experiment using unmodified carbon paper as the working electrode and NrGO on carbon paper as the counter electrode in a single-cell configuration. Short-circuited current and mixed potential at a periodic switch between Ar, CO (0.04 atm), O_2_ (0.2 atm), and a mixture of CO (0.04 atm) + O_2_ (0.2 atm). Reaction conditions: 0.25 M PBS (15 mL, pH 7.2); 25 ± 1 °C.

1. In the absence of NrGO, but in the presence of Au NPs

In contrast, in the absence of NrGO but with Au NPs present, no appreciable short-circuited current was detected at an O_2_ atmosphere (as shown in **Fig. S8**), highlighting the absence of a driving force from ORR for an oxidation half-reaction on the Au NPs or carbon paper. **Figure S8** shows near-zero currents (within ± 0.5 µA) across all feed conditions, i.e., Ar, O_2_, CO and CO+O­_2_ atmosphere, demonstrating that NrGO is essential for electron transfer between the anode and cathode.

Additionally, the mixed potential remained unchanged when switching feeds from Ar to CO and from O_2_ to CO+O_2_. However, the introduction of O_2_ led to a positive shift of mixed potential and a negative value of short-circuited current. This behavior was speculated to be attributed to the couple of a cathodic reaction (ORR) on Au NPs side and an anodic reaction (carbon corrosion) on blank carbon paper side. Due to the sluggish nature of carbon corrosion, the short-circuited current was minimal and the mixed potential was close to the equilibrium potential of ORR on Au NPs.


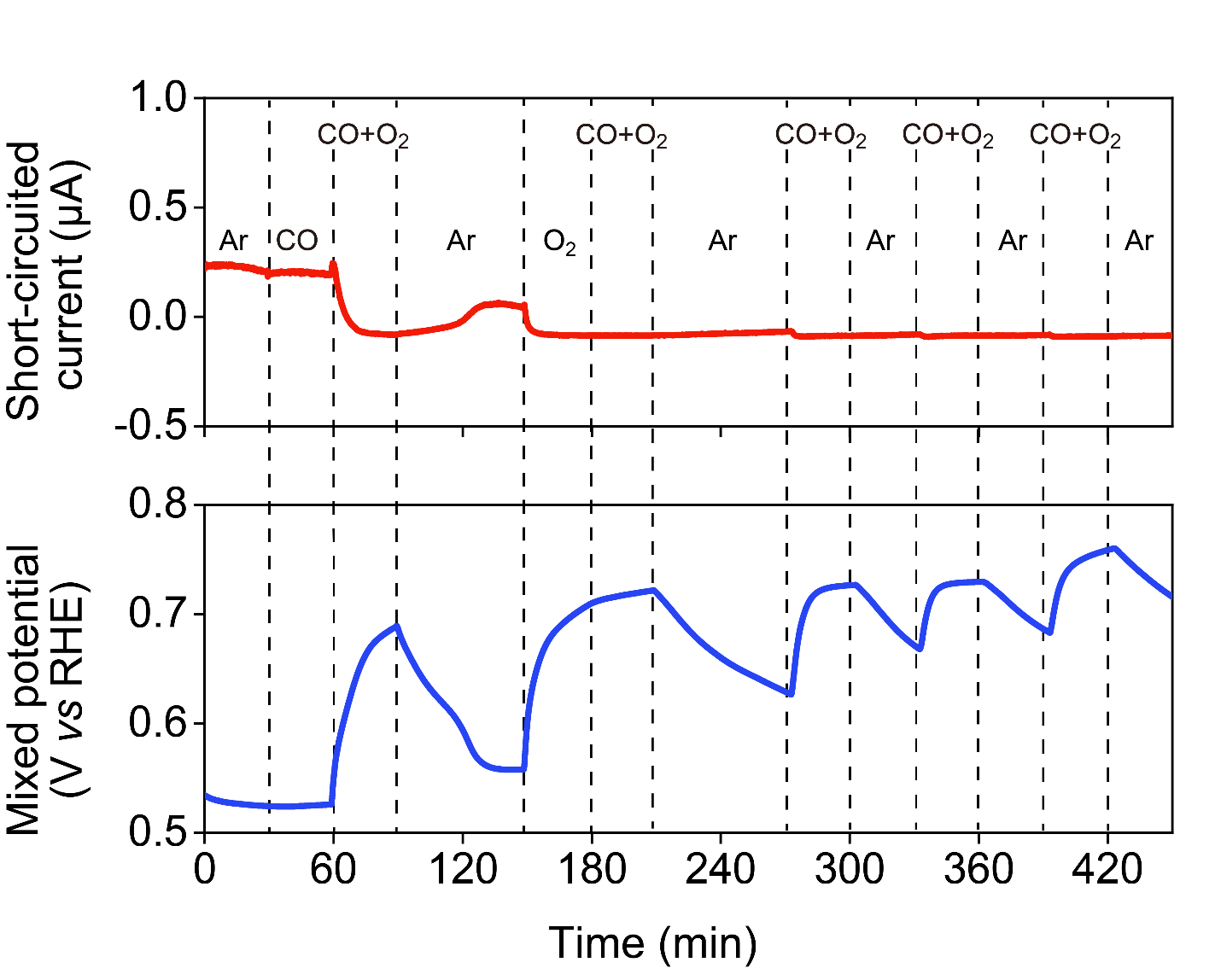


**Fig. S8**. Short-circuit current and mixed potential as a function of time in a control experiment using Au NPs on carbon paper as the working electrode and unmodified carbon paper as the counter electrode in a single-cell configuration. Short-circuited current and mixed potential at a periodic switch between Ar, CO (0.04 atm), O_2_ (0.2 atm), and a mixture of CO (0.04 atm) + O_2_ (0.2 atm). Reaction conditions: 0.25 M PBS (15 mL, pH 7.2); 25 ± 1 °C.

S3. Impact of mass transport and electrolyte resistance

**Figure S9** displays the Tafel behavior of electrochemical CO oxidation (COOR) on the Au electrode, derived from the current−potential data shown in **Fig. 3a**. It reveals a Tafel slope of 121 mV dec^−1^ in the kinetically controlled region, which is consistent with other reports.^[19–21]^ Then, the COOR on Au becomes diffusion controlled with the increase of anodic polarization.


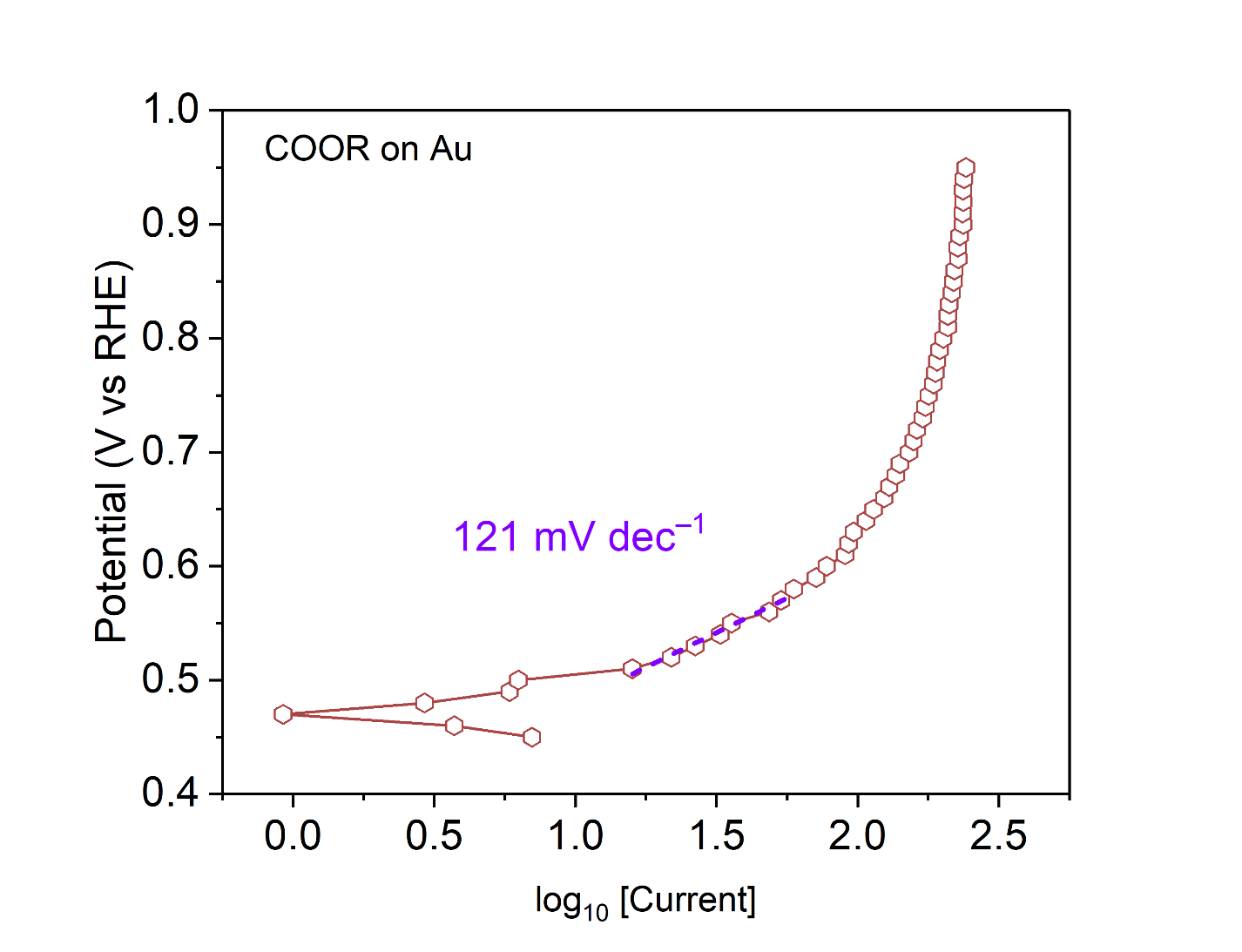


**Fig. S9**. Tafel plot for electrochemical CO oxidation on Au electrodes derived from the current−potential data shown in **Fig. 3a**.

Moreover, based on the prior reports on ORR over Au,^[19–21]^ a two-electron transfer pathway for ORR may also happen and produce the H_2_O_2_, as Equation (S3-2). Thus, the relevant half-reactions and overall CO oxidation can be represented as:

| $CO+H_{2}O\to CO_{2}+2H^{+}+2e^{-}$ | (S3-1) |
| --- | --- |
| $O_{2}+2H^{+}+2e^{-}\to H_{2}O_{2}$ | (S3-2) |
| $CO+O_{2}+H_{2}O\to CO_{2}+H_{2}O_{2}$ | (S3-3). |

Here, we merely highlight the potential for the H_2_O_2_ pathway. This aspect, however, falls outside the scope of the present study.

We then examined the impact of gas mass transport by using Au NPs deposited on fluorine-doped tin oxide (FTO) glass as the electrode (prepared following the same procedure as the gas diffusion electrodes, except with FTO replacing carbon paper). Unlike carbon paper, FTO does not have a gas diffusion layer, thereby reducing the CO and O_2_ flux to the Au NPs via a direct transport path between the reaction head-space and the catalyst film. The mixed-potential-driven reaction mechanism provides detailed insights into how the overall reaction is affected by the limitation on gas transport.

As shown in **Fig. S10**, inhibited gas transport significantly suppressed the short-circuited currents. Under an O_2_ atmosphere, a small short-circuited current was measured between the Au NPs and NrGO, while the mixed potential was approximately 0.75 V. Although the short-circuited currents were small, the shift in the mixed potential indicates that the intrinsic kinetics of Au NPs oxidation occurred in conjunction with ORR on the NrGO side. The blockage of CO transport to Au NPs resulted in almost no short-circuited current under the CO atmosphere, with the mixed potential remaining unchanged compared to an Ar feed. Furthermore, the addition of O_2_ to CO produced similar currents and mixed potentials to those observed in the O_2_-only atmosphere. This highlights the critical role of gas mass transport in mixed-potential-driven catalysis.


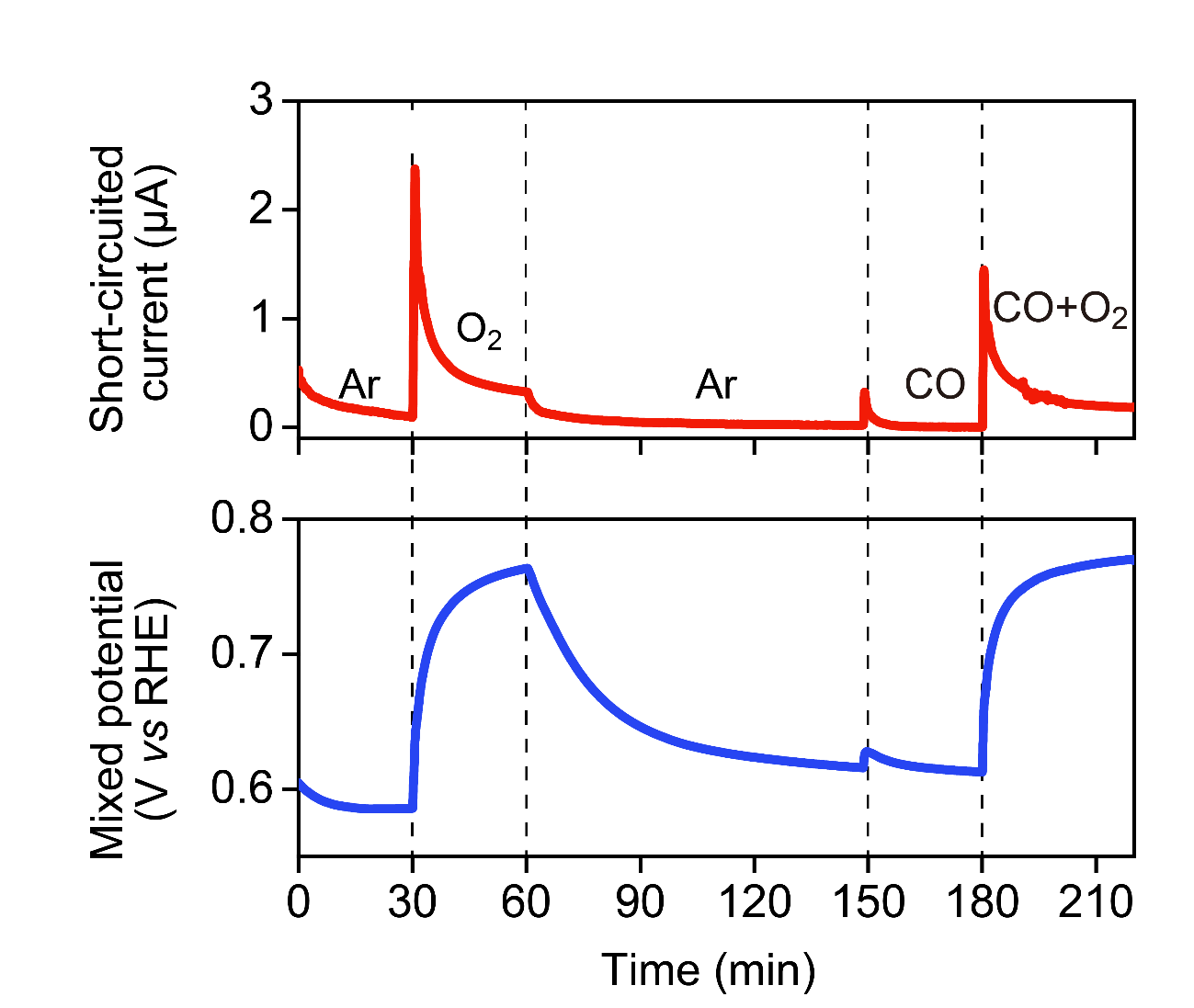


**Fig. S10**. Short-circuit current and mixed potential as a function of time in a control experiment using Au NPs on FTO (working electrode, without gas diffusion layer) and NrGO on carbon paper (counter electrode) in a single-cell configuration. Short-circuited current and mixed potential at a periodic switch between Ar, CO (0.04 atm), O_2_ (0.2 atm), and a mixture of CO (0.04 atm) + O_2_ (0.2 atm). Reaction conditions: 0.25 M PBS (15 mL, pH 7.2); 25 ± 1 °C.

In our model system, the two electrodes were spatially separated, which could result in the consideration of voltage drop over the electrolyte phase. The ohmic potential drop across the bulk electrolyte solution from the cathode to the anode calculated by Ohm’s law

| $\Delta\phi_{\mathrm{IR}}=R_{\mathrm{sol}}i$ | (S3-4) |
| --- | --- |

where $R_{\mathrm{sol}} (\Omega m^{2})$ is the areal resistence of the bulk solution, and $i$ is the current. The resistence of the bulk solution can be estimated by

| $R_{\mathrm{sol}}=\frac{l}{\kappa A}$ | (S3-5) |
| --- | --- |

where $l (\mathrm{cm})$ is the distance between the cathode and the anode, $\kappa(S cm^{-1} )$ is ion conductivity of the electrolyte, and $A (\mathrm{cm}^{2})$ is the effective cross-sectional area of the electrodes. The ion conductivity of for 0.156 M PBS is 0.013 ($S cm^{-1}$).^[22]^ Assuming approximately linear dependence of conductivity on concentration, we can estimate that the conductivity of 0.25 M PBS is 0.0208 ($S cm^{-1}$). In our case

| $\phi_{\mathrm{IR}}=\frac{l}{\kappa A}i=\frac{1 (\mathrm{cm})}{0.0208 \left( S cm^{-1} \right)\times1(\mathrm{cm}^{2})}\times1\times{10}^{-4} (A)=4.8\times{10}^{-3} V$ | (S3-6) |
| --- | --- |

This estimated potential drop of ~4.8 mV is significantly smaller than the typical kinetic overpotentials (> 100 mV) required to drive the anodic and cathodic half-reactions in a mixed potential system. The current density seems to be small enough to ensure that the electric potential drop is negligible over the solution phase.

However, we emphasize that this effect becomes increasingly important when the ionic conductivity is limited or current is larger. To investigate the importance of ion conductivity in the electrochemical experiments, pure water rather than PBS was used as electrolyte. Almost no reaction current was detected as shown in **Fig. S11**.


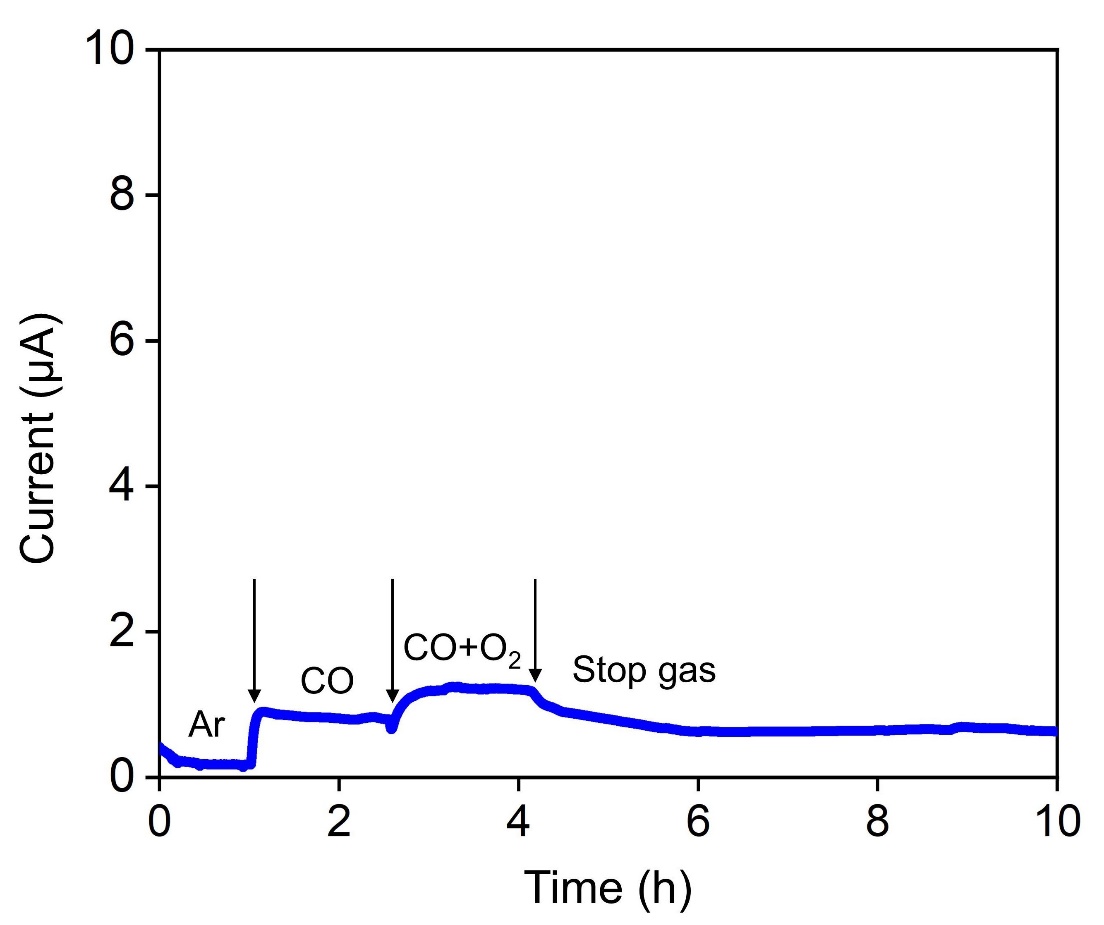


**Fig. S11**. A control experiment for water used as the reaction media. Reaction conditions: Au NPs on carbon paper as the working electrode and NrGO on carbon paper as the counter electrode; pure water as the electrolyte (15 mL); 25 ± 1 °C; purge-gas: Ar, CO (0.04 atm), and a mixture of CO (0.04 atm) + O_2_ (0.2 atm); total flow rate: 50 cm^–3^ min^–1^.

S5. Short-circuited experiments in the H-cell

In the mixed-potential-driven catalysis, the position of mixed potential approaches the equilibrium potential of the kinetically favored half-reaction.^[23]^ Notably, the mixed potential for a gas mixture of CO+O_2_ lies between the values observed in O_2_-only and CO-only atmospheres. During operation, the mixed potential shifted positively to 0.82 V in an O₂ atmosphere and negatively to 0.59 V in a CO atmosphere (**Fig. 2b**), consistent with the current–potential behaviors of ORR and COOR (**Fig. 3a**).

We further investigated this phenomenon using an H-cell. The two compartments of the H-cell (each with a volume of 30 mL) were separated by a sintered glass frit to prevent bulk mixing of liquids and gases. To identify the electrochemical half-reactions occurring on each side, different gases were introduced separately into the Au NPs compartment and the NrGO compartment. It is important to note that the short-circuited experiments conducted in the H-cell are not mixed-potential-driven reactions. In the H-cell setup, the anode and cathode reactions are manually controlled by introducing different reactants to the electrodes, similar to a fuel cell. In contrast, in actual mixed-potential-driven reactions, the catalyst components are exposed to the same reactant environment. The half-reactions that occur are governed by the intrinsic catalytic activity of each component. In other words, the catalyst materials ‘select’ which reactions to catalyze based on their relative activities and selectivities.

Before the experiment, both compartments were sparged with Ar gas for over 30 minutes at a of flow rate of 50 cm^3^ min^−1^ to ensure removal of the residual O_2_. The results are discussed below (**Fig. S12**).


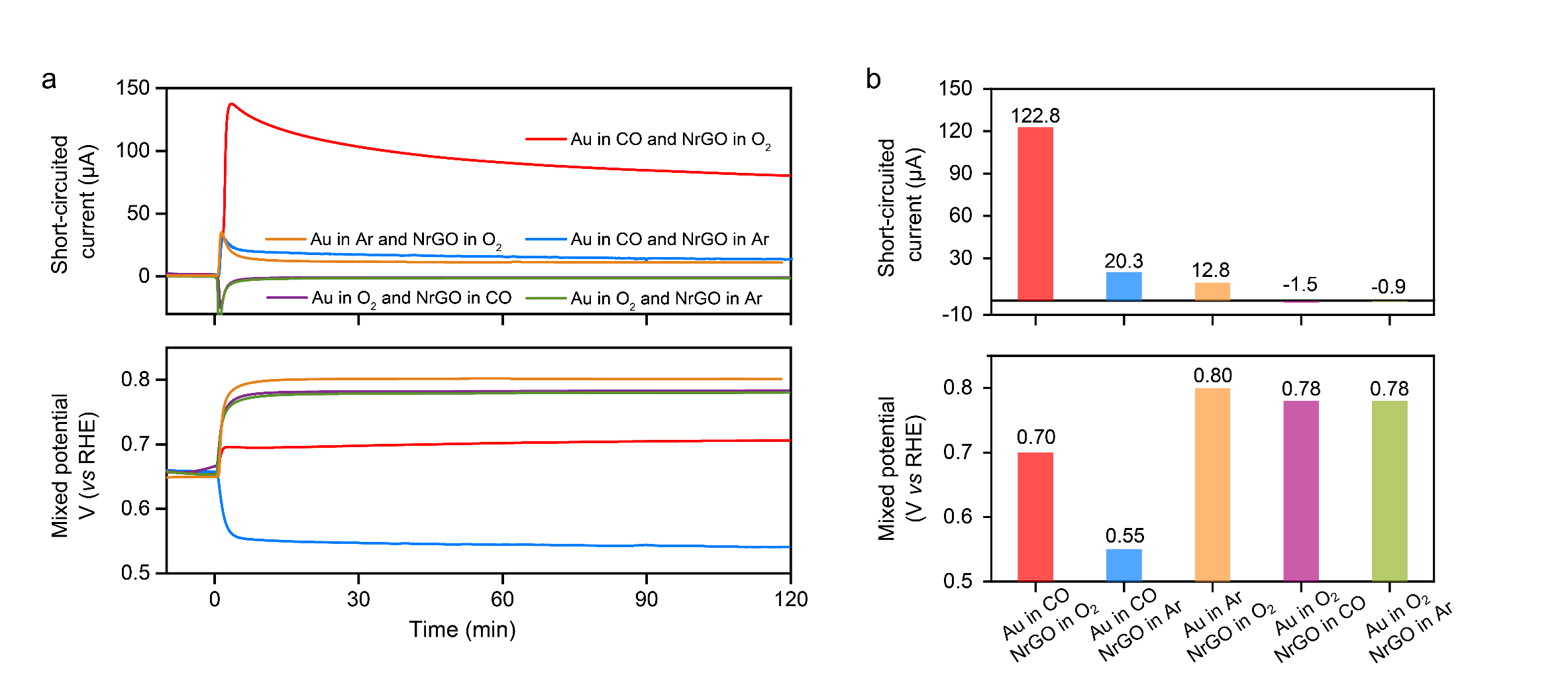


**Fig. S12**. Short-circuited currents and mixed potentials in the H-cell. Reaction conditions: 0.25 M PBS (pH 7.2, 10 mL in each compartment); 25 ± 1 °C; working electrode: Au NPs; counter electrode: NrGO. Gas flow was separately introduced into the two chambers, with Au NPs and NrGO exposed to different atmospheres. The partial pressures of CO and O_2_ were 0.04 atm and 0.2 atm, respectively, under all feeding conditions.

(1) CO in Au NPs compartment, O_2_ in NrGO compartment.

The independent current–potential behaviors of COOR and ORR on Au NPs and NrGO suggest that Au NPs are responsible for carrying out both COOR and ORR, while NrGO supports only ORR. In this case, the observed short-circuited current via the external circuit in the single cell is given by: $i^{\mathrm{Single}}=\left| i_{\mathrm{Au}}^{\mathrm{COOR}}|-|i_{\mathrm{Au}}^{\mathrm{ORR}} \right|=\left| i_{\mathrm{NrGO}}^{\mathrm{ORR}} \right|$. If this is true, one should expect a larger external short-circuited current in the H-cell: $i^{H-cell}=\left| i_{\mathrm{Au}}^{\mathrm{COOR}} \right|=\left| i_{\mathrm{NrGO}}^{\mathrm{ORR}} \right|$, when CO and O_2_ are separately introduced into Au NPs side and NrGO compartments, respectively. When CO was bubbled into the Au NPs compartment and O_2_ in the NrGO compartment, a higher short-circuited current of approximately 122.8 μA was observed (compared to the single cell), with the measured mixed potential at 0.71 V. This result is verified by the fact that COOR and ORR occur on the Au NPs and NrGO side, respectively, as shown in **Fig. S13**.


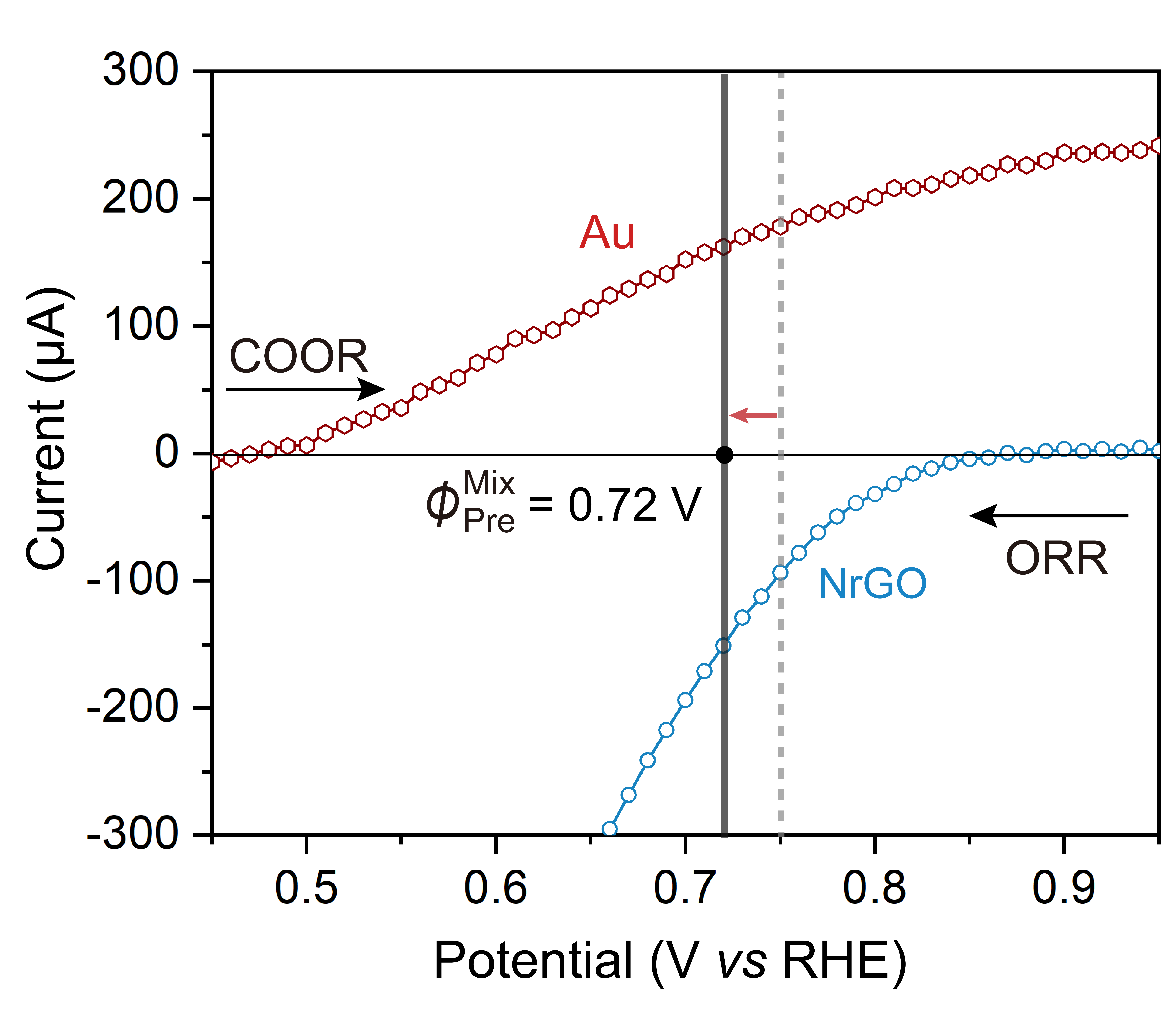


**Fig. S13.** The COOR and ORR occur on the Au NPs and NrGO side, respectively, resulting in a mixed potential of 0.72 V and the current of 151 μA.

(2) CO in Au NPs compartment, Ar in NrGO compartment.

A positive current of 20.3 μA indicates that an electrochemical reduction reaction occurred on NrGO, coupled with CO oxidation on Au NPs. In mixed-potential-driven catalysis, the mixed potential approaches the equilibrium potential of the kinetically favored half-reaction. Since the kinetics of CO oxidation are more favorable than those of NrGO reduction, the mixed potential was observed near the equilibrium potential for CO oxidation on Au NPs, at 0.55 V.

(3) Ar in Au NPs compartment, O_2_ in NrGO compartment.

A smaller positive current of 12.8 μA indicates that Au NPs oxidation occurred, driven by ORR on NrGO. The mixed potential of 0.80 V suggests that the intrinsic kinetics of Au NPs oxidation are significantly slower than those of ORR on NrGO.

(4) O_2_ in Au NPs compartment, Ar or CO in NrGO compartment.

The short-circuited current was negative and very small, which could be even neglected. Moreover, the mixed potential located at 0.78 V at both cases. It means that the oxidation reaction on NrGO side (both COOR and the self-oxidation of NrGO) are rather difficult to happen.

S6. Calibration processes

The CO oxidation was conducted as PBS buffer (pH 7.2), where CO_2_ was trapped in solution through the formation of carbonate (CO_3_^2−^), bicarbonate (HCO_3_^−^), dissolved CO_2(aq)_ and carbonic acid (H_2_CO_3_). The relative proportions of CO_3_^2−^, HCO_3_^−^, CO_2(aq)_, and H_2_CO_3_ depend upon the temperature and the solution pH. The carbonate system in aqueous involves the following equilibrium reactions.

| $\mathrm{CO}_{2\left( \mathrm{aq} \right)}+H_{2}O\underset{\leftrightarrow}{K_{H}}H_{2}CO_{3}$ | (S6-1) |
| --- | --- |
| $H_{2}CO_{3} \underset{\leftrightarrow}{K_{1}} {\mathrm{HCO}_{3}}^{-}+H^{+} \mathrm{or} \mathrm{CO}_{2\left( \mathrm{aq} \right)}+H_{2}O \underset{\leftrightarrow}{K_{1}} {\mathrm{HCO}_{3}}^{-}+H^{+}$ | (S6-2) |
| ${\mathrm{HCO}_{3}}^{-} \underset{\leftrightarrow}{K_{2}} {\mathrm{CO}_{3}}^{2-}+H^{+}$ | (S6-3) |

In Eq. S1, the hydration equilibrium constant between CO_2(aq)_ and H_2_CO_3_ is small ($K_{H}\approx{10}^{-2.8}$ at 25°C), meaning the conversion of CO_2(aq)_ to H_2_CO_3_ is slow, particularly in pure systems at pH below 8.^[24]^ Consequently, most of the undissociated dissolved CO_2_ remains in the form of CO_2(aq)_ rather than H_2_CO_3_. On the other hand, at high pH, the reaction between CO_2(aq)_ and hydroxide to form bicarbonate is relatively rapid. Thus, when describing the dissociation constants for the carbonic acid system in Equation (S6-1) and (S6-2), a single term can be used to represent the concentrations of both species CO_2(aq)_ and H_2_CO_3_. For simplicity, the sum of the concentrations of CO_2(aq)_ and H_2_CO_3_ is denoted as [CO_2(aq)_]. The first dissociation constant of carbonic acid is given by

| $K_{1}=\frac{\left[ {\mathrm{HCO}_{3}}^{-} \right]\left[ H^{+} \right]}{\left[ CO_{2(\mathrm{aq})} \right]}$ | (S6-4) |
| --- | --- |

and the second dissociation constant is

| $K_{2}=\frac{\left[ {\mathrm{CO}_{3}}^{2-} \right]\left[ H^{+} \right]}{\left[ {\mathrm{HCO}_{3}}^{-} \right]}$ | (S6-5) |
| --- | --- |

We can assume that the total carbonate concentration is

| $C_{T}=\left[ CO_{2(\mathrm{aq})} \right]+\left[ {\mathrm{HCO}_{3}}^{-} \right]+\left[ {\mathrm{CO}_{3}}^{2-} \right]$ | (S6-6) |
| --- | --- |

where activity coefficients are equal to one. With the electrical neutrality condition,

| $\left[ H^{+} \right]=\left[ OH^{-} \right]+\left[ {\mathrm{HCO}_{3}}^{-} \right]+2\left[ {\mathrm{CO}_{3}}^{2-} \right]$ | (S6-7) |
| --- | --- |

and the acid base relationship between the proton and hydroxide,

| $\left[ H^{+} \right]\left[ OH^{-} \right]=K_{w}={10}^{-14}$ | (S6-8) |
| --- | --- |

the fraction of each species can be expressed in terms of $\left[ H^{+} \right]$, $K_{1}$, and $K_{2}$:

| $\frac{\left[ CO_{2(\mathrm{aq})} \right]}{C_{T}}=\frac{\left[ H^{+} \right]^{2}}{\left[ H^{+} \right]^{2}+K_{2}\left[ H^{+} \right]+K_{1}K_{2}}$ | (S6-9) |
| --- | --- |
| $\frac{{[\mathrm{HCO}_{3}}^{-}]}{C_{T}}=\frac{\left[ H^{+} \right]K_{2}}{\left[ H^{+} \right]^{2}+K_{2}\left[ H^{+} \right]+K_{1}K_{2}}$ | (S6-10) |
| $\frac{\left[ {\mathrm{CO}_{3}}^{2-} \right]}{C_{T}}=\frac{K_{1}K_{2}}{\left[ H^{+} \right]^{2}+K_{2}\left[ H^{+} \right]+K_{1}K_{2}}$ | (S6-11) |

In particular, at a temperature of 25°C and zero ionic strength, $K_{1}={10}^{-6.35} \left( pK_{1}=6.35 \right)$, $K_{2}={10}^{-10.33} \left( pK_{2}=10.33 \right).$^[25]^ These carbonate equilibria can be used to estimate the concentrations of dissolved species as a function of solution pH, as shown in **Fig. S14**.


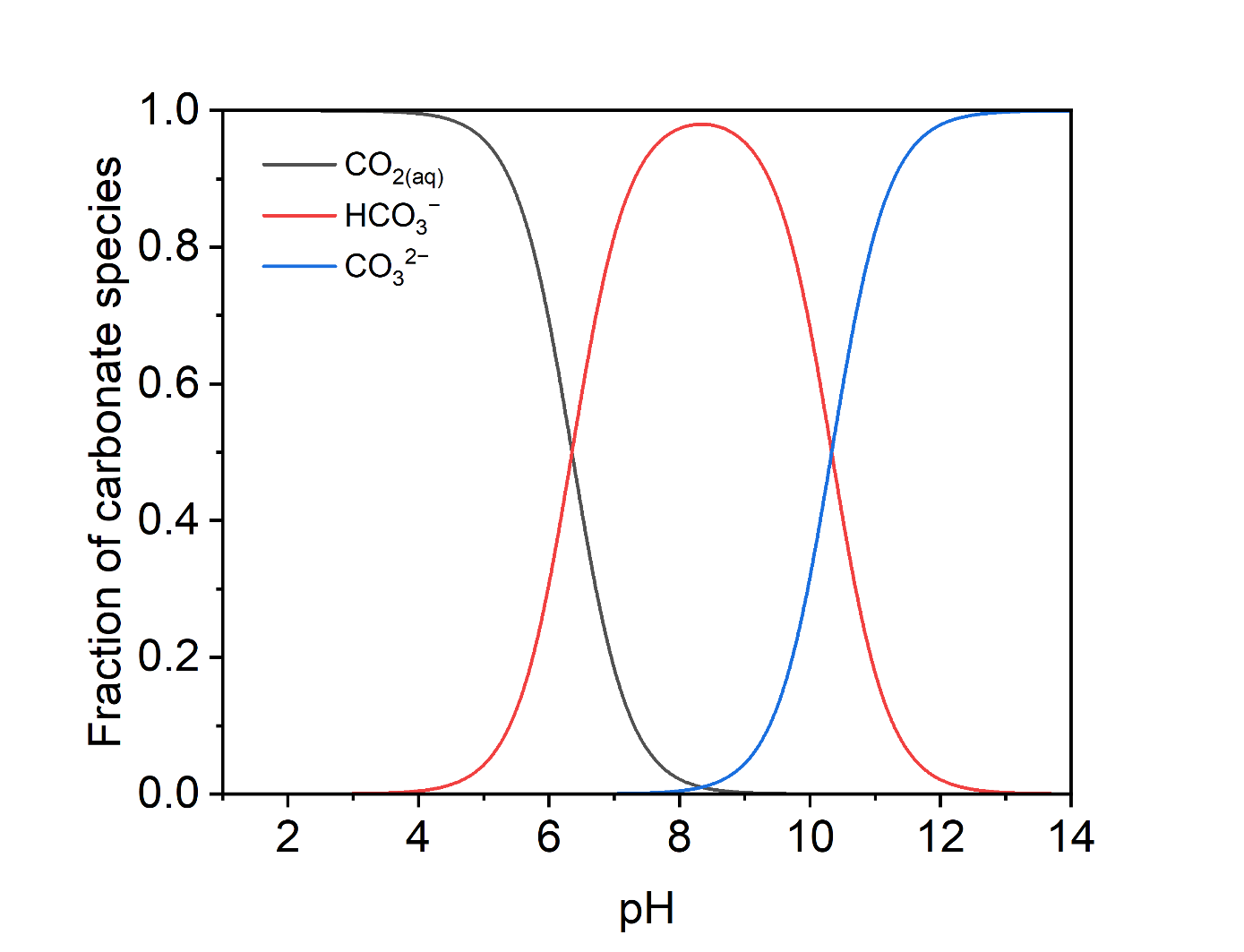


**Fig. S14**. The distribution of carbonate species as a fraction of the total dissolved carbonate in relation to solution pH.

We prepared the standard solutions of defined HCO_3_^−^ concentrations ([HCO_3_^−^]_Defined_) by using buffer (pH 7.2, same as the reaction electrolyte) as the substrate. The [HCO_3_^−^]_Defined_ represents the concentration of total carbonate species [*C*]_Tot_. For example, a 10 mM [HCO_3_^−^]_Defined_ solution was prepared by dissolving 1.0012 g KHCO_3_ (molecular weight of KHCO_3_ = 100.12 g/mol) in the buffer and diluting to 1 L in a volumetric flask. Other standard [HCO_3_^−^]_Defined_ solutions were prepared by further diluting the buffer. Principally, the defined HCO_3_^−^ concentration will equilibrate to its equilibrium concentration ([HCO_3_^−^]_Eq_) at a specific pH, as described by **Fig. S14**.


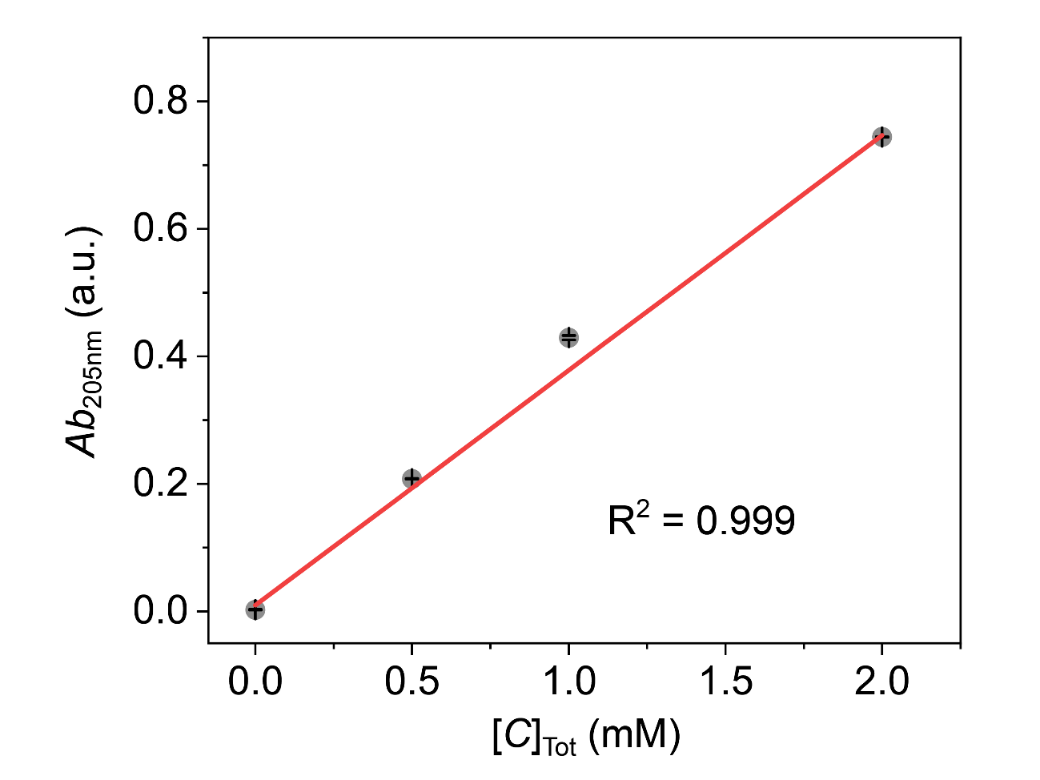


**Fig. S15**. Calibration curve of bicarbonate for UV–vis absorbance at 205 nm. The slope returns a molar absorptivity coefficient of 368.7 M^−1^ cm^−1^. All spectra and baselines collected in 0.25 M phosphate buffer solution (pH 7.2).

Then, the HCO_3_^−^ concentrations detected by UV–vis ([HCO_3_^−^]_Detected_) correspond to the equilibrium concentration [HCO_3_^−^]_Eq_. At pH 7.2, we have

| $\left[ {\mathrm{HCO}_{3}}^{-} \right]_{D\mathrm{etected}}=\left[ {\mathrm{HCO}_{3}}^{-} \right]_{\mathrm{Eq}}=0.876\times\left[ C \right]_{\mathrm{Tot}}=0.876\times\left[ {\mathrm{HCO}_{3}}^{-} \right]_{\mathrm{Defined}}$ | (S6-12) |
| --- | --- |

Specifically, UV–vis absorbance at 205 nm served as an effective surrogate for monitoring bicarbonate levels ^[5–7]^, which obeys the linear behavior of Beer’s Law (**Fig. S15**). Here, we use the [*C*]_Tot_ as the scale. The slope returns a molar absorptivity coefficient of 368.7 M^−1^ cm^−1^.

Also, since our experiments were conducted in a buffer (pH = 7.2), the detected [HCO_3_^−^]_Detected_ is part of the total product of CO oxidation, [*C*]_Tot,exp_.

| $\left[ {\mathrm{HCO}_{3}}^{-} \right]_{D\mathrm{etected}}=0.876\times\left[ C \right]_{Tot,exp} \left( at pH=7.2 \right)$ | (S6-13) |
| --- | --- |

Therefore, combining Equation (S6-12) and (S6-13), we conclude that the total product total product of CO oxidation, [*C*]_Tot,exp_ is equal to the prepared defined [HCO_3_^−^]_Defined_ from the calibration curve in **Fig. S15**.

| $\left[ C \right]_{Tot,exp}=\left[ {\mathrm{HCO}_{3}}^{-} \right]_{\mathrm{Defined}}=\left[ C \right]_{\mathrm{Tot}}$ | (S6-14) |
| --- | --- |

S7. Product yield estimation for non-short-circuited and short-circuited conditions

For the short-circuited condition, the system adopts a mixed potential of 0.75 V (**Fig. 3**). The mixed current, represented the total reaction rate, is: $i_{\mathrm{mix}}^{\mathrm{SC}}= \left| i_{\mathrm{Au}}^{\mathrm{COOR}}|=|i_{\mathrm{Au}}^{\mathrm{ORR}} \right|+\left| i_{\mathrm{NrGO}}^{\mathrm{ORR}} \right|=176 \mu A$. Disconnecting the external wire (non‑short‑circuited condition) results in Au NPs electrode to establish its own local mixed potential. These non-short-circuited experiments were performed under the same reaction conditions as the short-circuited experiments, with the only difference being the absence of an electrical connection between the Au NPs and NrGO electrodes. The mixed potential for Au electrode alone is 0.72 V and the mixed current, $i_{\mathrm{mix}}^{n.SC}$, is 160 μA, as shown in **Fig. S16**.


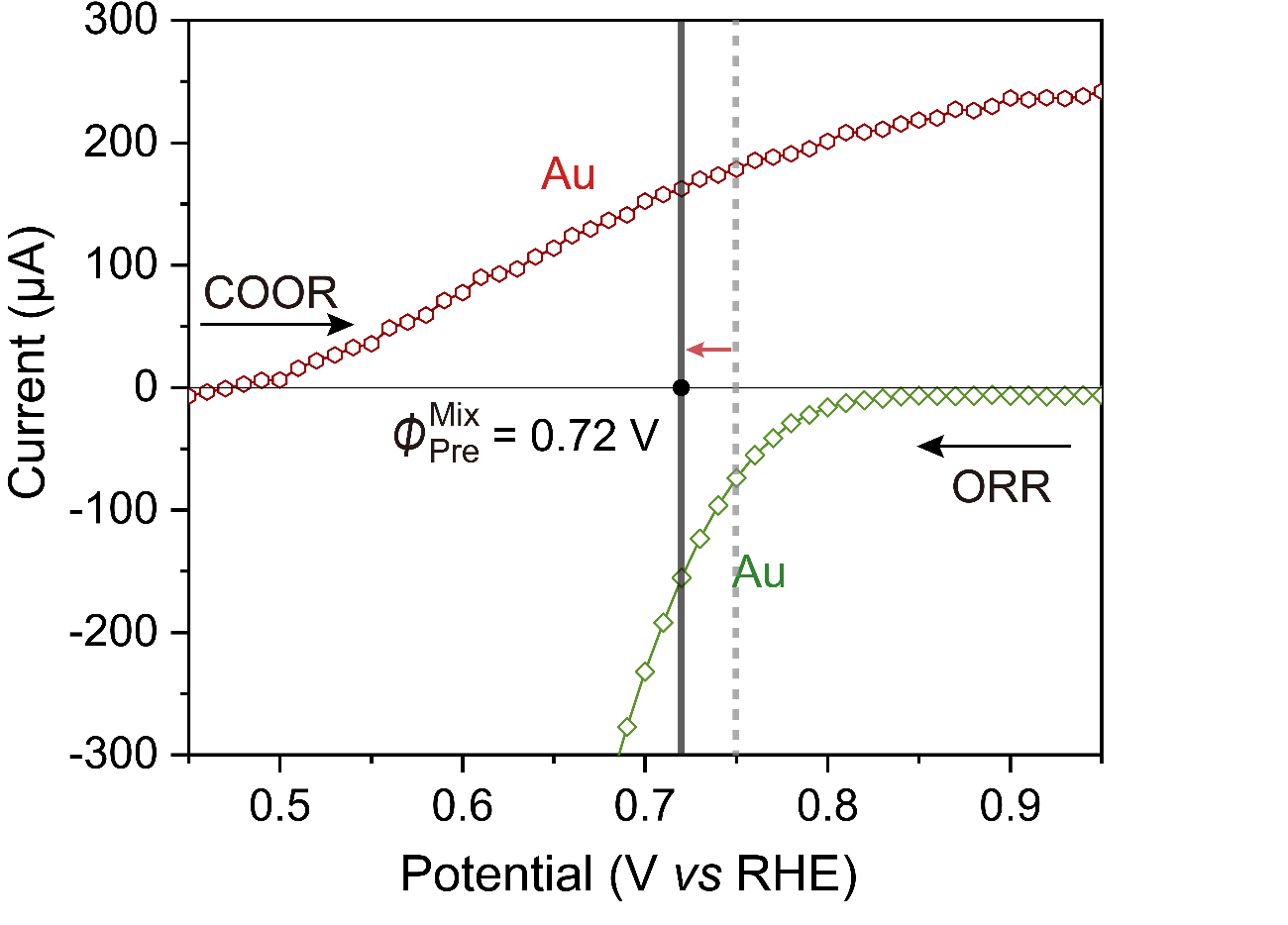


**Fig. S16.** The local mixed-potential-driven CO oxidation on Au NPs only. The mixed potential for Au electrode alone is 0.72 V and the mixed current, $i_{\mathrm{mix}}^{n.SC}$, is 160 μA.

Then, $N^{Pre.SC}$ and $N^{n.SC}$ represent the predicted amount of CO_2_-derived products under the short-circuited condition and the amount product measured by UV-vis under non-short-circuited conditions respectively. Assuming the amount of products is proportional to the current. The following relationship holds

| $\frac{N^{Pre.SC}-N^{n.SC}}{N^{n.SC}}=\frac{i_{\mathrm{mix}}^{\mathrm{SC}}-i_{\mathrm{mix}}^{n.SC}}{i_{\mathrm{mix}}^{n.SC}}=\frac{176-160}{160}=0.1$ | (S7-1) |
| --- | --- |

This relationship indicates that the ratio of CO_2_-derived products formed by Au NPs alone to those formed by the Au NPs-NrGO pair is 10:1. But we have to admit that the analysis is based on the assumption, that is, all products formed under non-short-circuited conditions, $N^{n.SC}$, originate from the mixed-potential-driven mechanism on the Au NPs. With this assumption, we plotted a predicted line ($N^{Pre.SC}$, dot line in **Fig. 4a**) based on the value of $N^{n.SC}$, that is, $N^{Pre.SC}=N^{n.SC}+0.1\times N^{n.SC}$. Here, the amount of CO_2_-derived products ($N^{n.SC}$) under non-short-circuited condition is the reference.

After short-circuiting, the increase amount products should match the electrons passed through the external circuit. That is, we could expect a two-electron stoichiometry between $N^{\mathrm{SC}}-N^{n.SC}$ and electrons number passed through the external circuit under short-circuited condition. Thus, a series of additional short-circuited experiments were conducted under a CO+O_2_ feed to assess the products at 1 h, 3 h, and 5 h, as shown in **Fig. S17**. The total molar electron transfer between the electrodes was calculated by integrating the current flow.


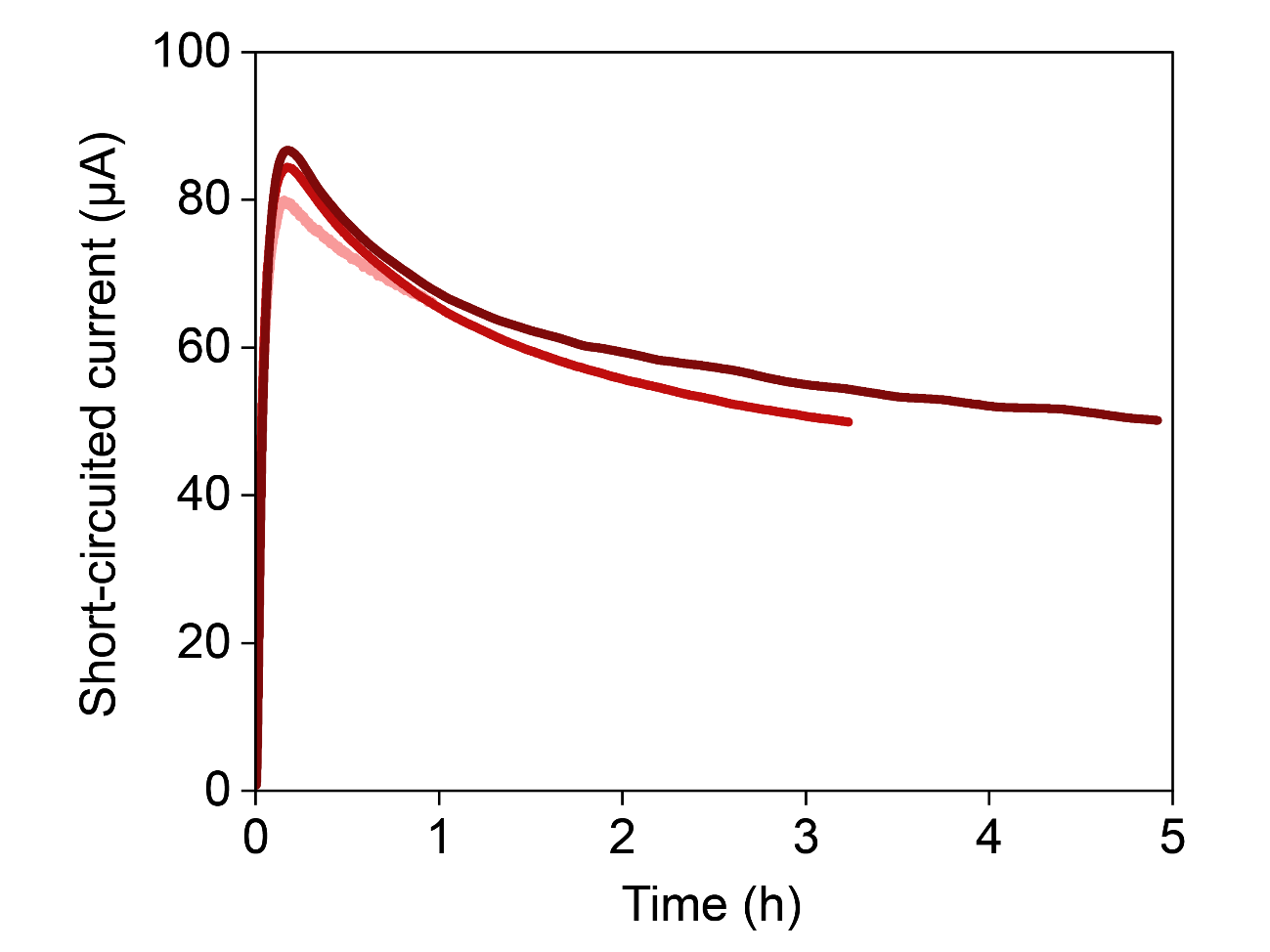


**Fig. S17**. Short-circuited currents under CO+O_2_ feed generated at different reaction time to estimate electrons passed through the external circuit. Reaction conditions: 0.25 M phosphate buffer solution (15 mL, pH 7.2); 25 ± 1 °C; CO (0.04 atm) + O_2_ (0.2 atm); total flow rate: 50 cm^–3^ min^–1^.

Then, we quantified the products for short-circuited condition ($N^{\mathrm{SC}}$) using UV–vis spectroscopy, as shown in **Fig. S18**. Background results for the Ar- and O_2_-purged solutions after approximately 1 hour were also included.


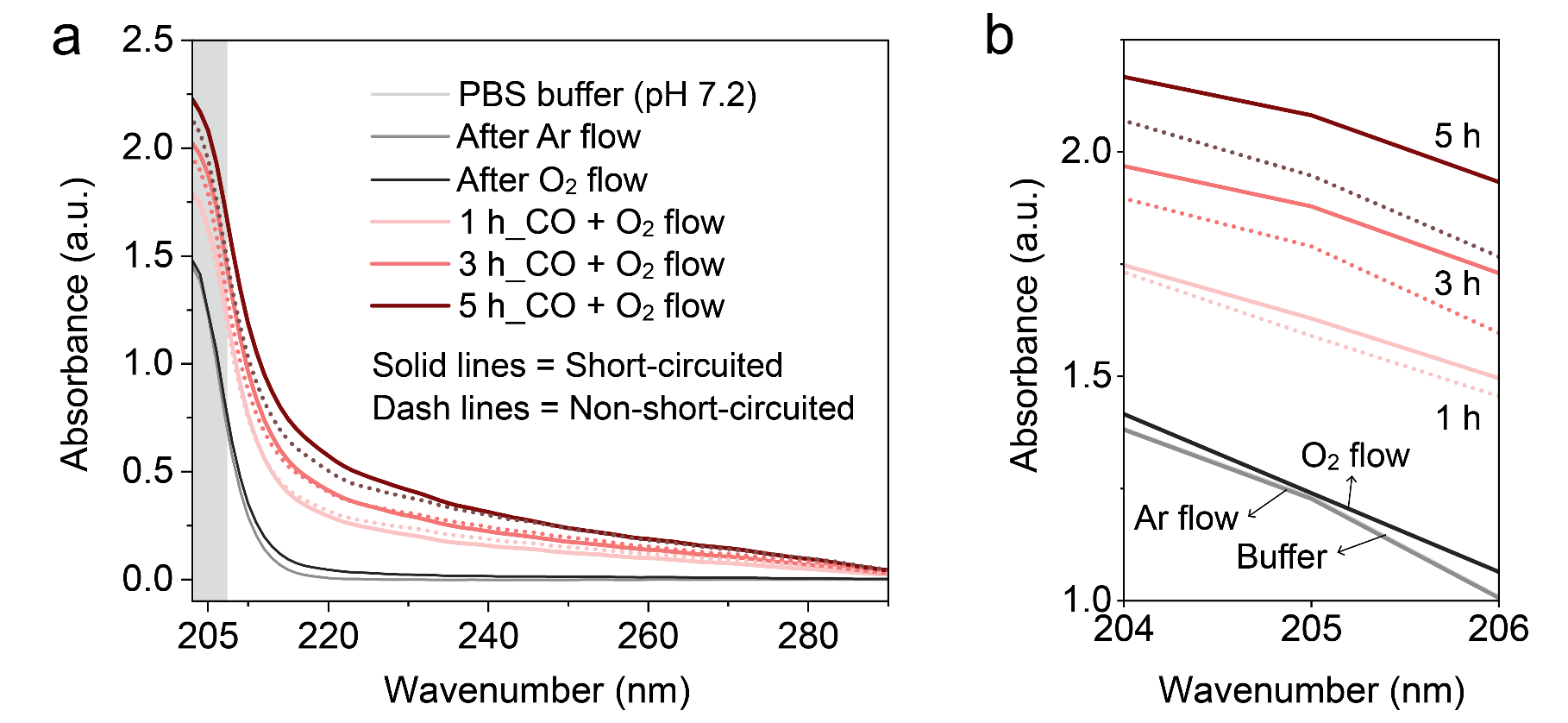


**Fig. S18**. (a) UV–vis spectra of the post-reaction solutions under different conditions. (b) Enlarged region from 204 to 206 nm in (a). The buffer lines are overlaid by those of the solutions after Ar flow. In both (a) and (b), solid lines represent short-circuited experiments, while dashed lines represent non-short-circuited experiments.

Additionally, a decrease in pH was expected if CO_2_ was captured in the alkaline solution. To test this, we conducted experiments at pH 11.9, as shown in **Fig. S19**. Upon switching the feed to CO+O_2_, a marked increase in short-circuited current was observed, indicating reaction suppression associated with the decreasing pH. Also, the mixed potential gradually rose from −0.113 V to −0.0177 V (*vs* Ag/AgCl). After approximately 1 h, the pH had decreased to 10.3, providing the evidence of the capture of product CO_2_ during operation.


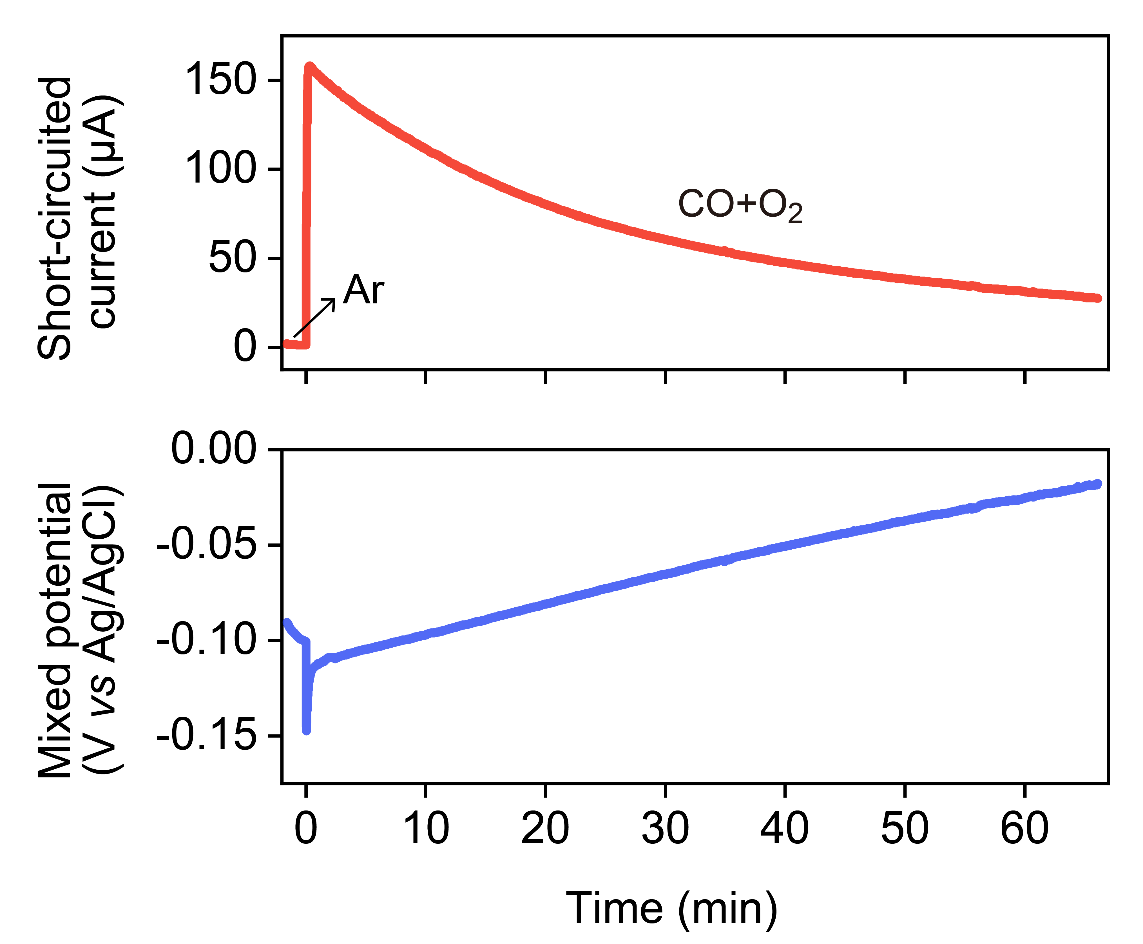


**Fig. S19**. An additional short-circuited experiment starting at pH 11.9 (10^−2^ M NaOH). After approximately 1 h, the pH decreased to 10.3. Reaction conditions: 25 ± 1 °C; CO (0.04 atm) + O_2_ (0.2 atm); total flow rate: 50 cm^–3^ min^–1^.

S8. Measured mixed potential versus pH

**Figure S20** shows the measured mixed potential of CO oxidation in the aqueous phase under a mixture of CO+O_2_ feed as a function of pH. It is important to note that there is no fundamental difference between **Fig. S20a** (versus SHE) and **b** (versus RHE); they are simply two different ways of presenting the same result.^[26]^ As the pH increases, the mixed potential shifts by −54 mV per pH unit (dashed line) versus the pH-independent SHE (**Fig. S20a**). This observed slope closely approximates the −59 mV per pH unit predicted by the Nernst equation, indicating a 1:1 proton-to-electron stoichiometry for CO oxidation at liquid phase. Provided that the potential is expressed versus pH-corrected RHE scale (**Fig. S20b**), the mixed potentials for all solutions nearly coincide, regardless of solution pH. The observation that the mixed potential scales Nernstianly with pH suggests that the bonding energetics and active site structure are invariant with respect to proton activity. This conclusion is valid within the pH range of 7.2 to 11.9.


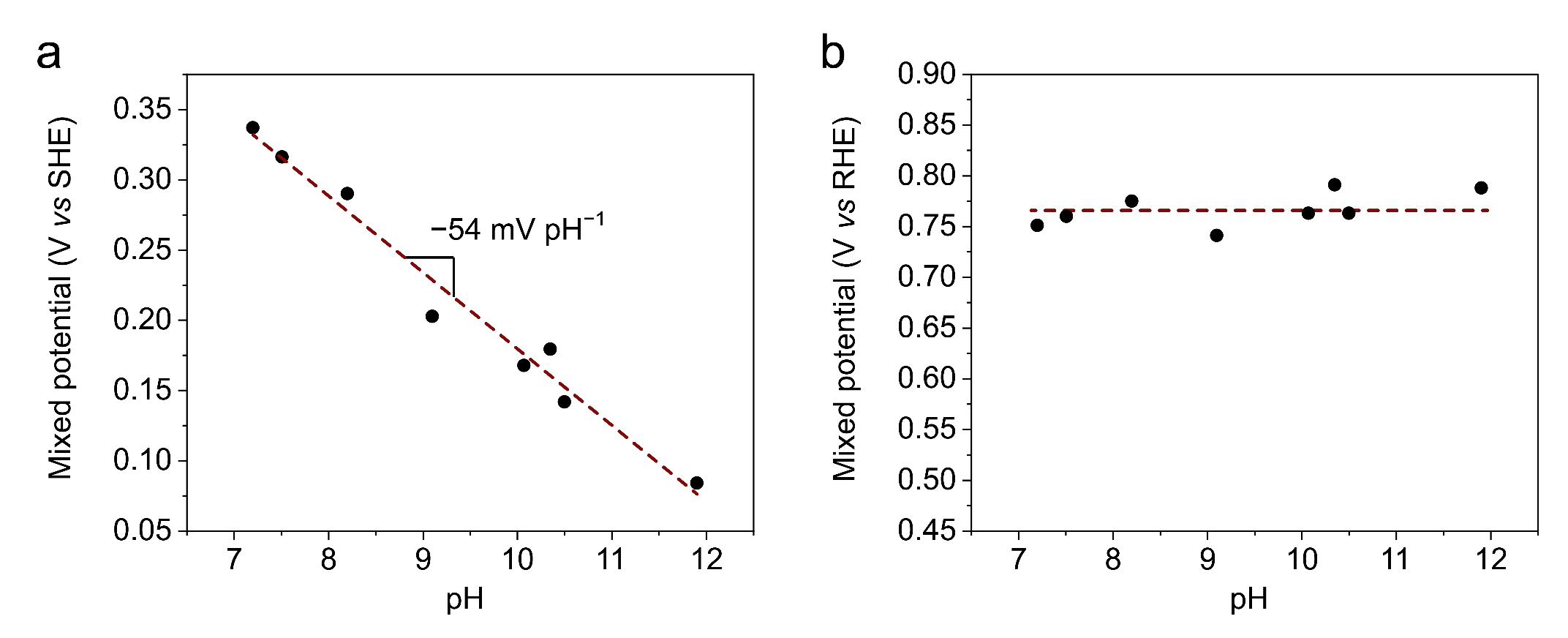


**Fig. S20**. Measured mixed potential of CO oxidation in the aqueous phase with a CO+O_2_ feed as a function of pH under the short-circuited condition, shown on the SHE scale (a) and the RHE scale (b). Reaction conditions: 25 ± 1 °C; CO (0.04 atm) + O_2_ (0.2 atm); total flow rate: 50 cm^–3^ min^–1^.

References

[1] S. K. Singh, K. Takeyasu, K. Homma, S. Ito, T. Morinaga, Y. Endo, M. Furukawa, T. Mori, H. Ogasawara, J. Nakamura, *Angewandte Chemie* **2022**, *134*, DOI 10.1002/ange.202212506.

[2] Y. Hatakeyama, K. Onishi, K. Nishikawa, *RSC Adv* **2011**, *1*, 1815–1821.

[3] H. Wender, L. F. De Oliveira, P. Migowski, A. F. Feil, E. Lissner, M. H. G. Prechtl, S. R. Teixeira, J. Dupont, *Journal of Physical Chemistry C* **2010**, *114*, 11764–11768.

[4] P. Peljo, M. D. Scanlon, A. J. Olaya, L. Rivier, E. Smirnov, H. H. Girault, *Journal of Physical Chemistry Letters* **2017**, *8*, 3564–3575.

[5] T. R. Martz, H. W. Jannasch, K. S. Johnson, *Mar Chem* **2009**, *115*, 145–154.

[6] J. Birkmann, C. Pasel, M. Luckas, D. Bathen, *Water Pract Technol* **2018**, *13*, 879–892.

[7] S. Bavarella, A. Brookes, A. Moore, P. Vale, G. Di Profio, E. Curcio, P. Hart, M. Pidou, E. J. Mcadam, *J Memb Sci* **2020**, *599*, 117682.

[8] J. Saavedra, H. A. Doan, C. J. Pursell, L. C. Grabow, B. D. Chandler, *Science (1979)* **2014**, *345*, 1599–1602.

[9] M. Ojeda, B. Z. Zhan, E. Iglesia, *J Catal* **2012**, *285*, 92–102.

[10] T. Fujitani, I. Nakamura, *Angewandte Chemie* **2011**, *123*, 10326–10329.

[11] J. T. Calla, R. J. Davis, *Journal of Physical Chemistry B* **2005**, *109*, 2307–2314.

[12] J. T. Calla, R. J. Davis, *Catal Letters* **2005**, *99*, 21–26.

[13] S. Zhao, F. Chen, S. Duan, B. Shao, T. Li, H. Tang, Q. Lin, J. Zhang, L. Li, J. Huang, N. Bion, W. Liu, H. Sun, A. Q. Wang, M. Haruta, B. Qiao, J. Li, J. Liu, T. Zhang, *Nat Commun* **2019**, *10*, 1–9.

[14] W. C. Ketchie, M. Murayama, R. J. Davis, *Top Catal* **2007**, *44*, 307–317.

[15] W. C. Ketchie, Y. L. Fang, M. S. Wong, M. Murayama, R. J. Davis, *J Catal* **2007**, *250*, 94–101.

[16] M. A. Sanchez-Castillo, C. Couto, W. B. Kim, J. A. Dumesic, *Angewandte Chemie - International Edition* **2004**, *43*, 1140–1142.

[17] F. M. Li, L. Huang, S. Zaman, W. Guo, H. Liu, X. Guo, B. Y. Xia, *Advanced Materials* **2022**, *34*, 1–20.

[18] S. G. Ji, H. Kim, W. H. Lee, H. S. Oh, C. H. Choi, *J Mater Chem A Mater* **2021**, *9*, 19834–19839.

[19] P. Rodriguez, D. Plana, D. J. Fermin, M. T. M. Koper, *J Catal* **2014**, *311*, 182–189.

[20] P. Rodriguez, N. Garcia-Araez, M. T. M. Koper, *Physical Chemistry Chemical Physics* **2010**, *12*, 9373–9380.

[21] S. C. Chang, A. Hamelin, M. J. Weaver, *Journal of Physical Chemistry* **1991**, *95*, 5560–5567.

[22] A. R. Kucernak, H. Wang, X. Lin, *ACS Energy Lett* **2024**, *9*, 3939–3946.

[23] M. Yan, N. A. P. Namari, J. Nakamura, K. Takeyasu, *Commun Chem* **2024**, *7*, 69.

[24] F. J. Millero, *Geochim Cosmochim Acta* **1995**, *59*, 661–677.

[25] F. J. Millero, D. Pierrot, K. Lee, R. Wanninkhof, R. Feely, C. L. Sabine, R. M. Key, T. Takahashi, *Deep Sea Research Part I: Oceanographic Research Papers* **2002**, *49*, 1705–1723.

[26] M. T. M. Koper, *Top Catal* **2015**, *58*, 1153–1158.
